# Burden and Correlates of Multiple Chronic Infections and Their Associations With Cancer Incidence in Chinese Adults: A Large Case‐Cohort Study

**DOI:** 10.1002/ijc.70555

**Published:** 2026-05-18

**Authors:** Ling Yang, Jonathan Clarke, Lea Kröller, Christiana Kartsonaki, Hannah Fry, Rima Jeske, Andrew Gordon, Sarah Clark, Michael Hill, Daniel Avery, Yiping Chen, Huaidong Du, Jun Lv, Dianjianyi Sun, Canqing Yu, Liming Li, Iona Y. Millwood, Tim Waterboer, Zhengming Chen, the China Kadoorie Biobank Collaborative Group, Junshi Chen, Zhengming Chen, Robert Clarke, Rory Collins, Liming Li, Jun Lv, Richard Peto, Robin Walters, Liming Li, Jun Lv, Canqing Yu, Dianjianyi Sun, Yuanjie Pang, Yuting Han, Can Hou, Qingmei Xia, Chao Liu, Pei Pei, Lang Pan, Xiao Han, Honglu Bian, Xinxin Chen, Daniel Avery, Maxim Barnard, Derrick Bennett, Ruth Boxall, Yiping Chen, Zhengming Chen, Jonathan Clarke, Robert Clarke, Huaidong Du, Ahmed Edris Mohamed, Hannah Fry, Prapthi Harish, Pek Kei Im, Andri Iona, Christiana Kartsonaki, Kshitij Kolhe, Hubert Lam, Kuang Lin, James Liu, Iona Millwood, Sam Morris, Qunhua Nie, Alfred Pozarickij, Maryam Rahmati, Paul Ryder, Maruf Sarder, Dan Schmidt, Becky Stevens, Iain Turnbull, Robin Walters, Baihan Wang, Lin Wang, Neil Wright, Ling Yang, Xiaoming Yang, Pang Yao, Zengchang Pang, Ruqin Gao, Kunzheng Lv, Shanpeng Li, Haiping Duan, Shaojie Wang, Yongmei Liu, Ranran Du, Liang Cheng, Xiaocao Tian, Hua Zhang, Dan Hu, Xiaoyan Zheng, Yujie Wang, Wei Sun, Shichun Yan, Chi Wang, Zhenyuan Wu, Lishun Zhai, Zhaoxi Pang, Shiwen Dong, Li Liu, Dapeng Yin, Bin He, Ying Liu, Xingren Wang, Tingting Ou, Xiangyang Zheng, Dewei Zheng, Shuai Yang, Lihui Li, Xingjiao Chen, Yan Xu, Jinyi Zhou, Ran Tao, Jian Su, Xikang Fan, Xuejia Chen, Yuxiao Huang, Yan Lu, Yujie Hua, Li Xing, Shuxian Wang, Jianrong Jin, Juping Ma, Jinchao Liu, Kaifei Zhu, Hongfu Ren, Xingfeng Shen, Ge Zhong, Wei Mao, Zhenzhen Lu, Yanxu Zhong, Lifang Zhou, Rong Pan, Jian Lan, Xiaoping Tan, Jinxue Tan, Yishan Xie, Liuping Wei, Liyuan Zhou, Sisi Wang, Xianping Wu, Ningmei Zhang, Xiaofang Chen, Xiaoyu Chang, Zhuo Wang, Yujin He, Mingqiang Yuan, Ling Wang, Xiaofang Chen, Zhaodong Wang, Qiang Sun, Yang Lin, Faqing Chen, Xiaolan Ren, Lijun Chang, Feiming Zhong, Jianjun Feng, Weijie Hu, Xiaofang Zhang, Yalin Chen, Honghong Wang, Jun Wang, Linqi Diao, Zhiwei Han, Dengjun Zhu, Kai Kang, Shixian Feng, Wenjie Yang, Huizi Tian, Yali Yan, Bing Han, Li Gao, Shaofang Li, Tianfang Xing, Wei Tang, Xiaolin Li, Huarong Sun, Xiaocong Zhao, Ying Li, Chen Hu, Pan He, Xukui Zhang, Yuanyuan Jin, Lan Luo, Min Yu, Ruying Hu, Hao Wang, Weiwei Gong, Jieming Zhong, Meng Wang, Chunxiao Xu, Keqing Gong, Hao Xu, Yuan Cao, Kaixu Xie, Lingli Chen, Xiaomei Tu, Junlong Pan, Xiaojun Li, Li Yin, Huilin Liu, Yuan Liu, Lei Yin, Xian Xie, Jing Wang, Bo Xiao, Zongwei Deng, Yuan Peng, Libo Zhang, Chan Qu, li Deng, Qili Jiang, Yanling Chen

**Affiliations:** ^1^ Clinical Trial Service Unit and Epidemiological Studies Unit (CTSU), Nuffield Department of Population Health University of Oxford Oxford UK; ^2^ Infections and Cancer Epidemiology Division, German Cancer Research Center (DKFZ) Heidelberg Germany; ^3^ Department of Epidemiology and Biostatistics, School of Public Health Peking University Health Science Centre Beijing China; ^4^ Peking University Center for Public Health and Epidemic Preparedness and Response, Peking University Beijing China; ^5^ Key Laboratory of Epidemiology of Major Diseases (Peking University), Ministry of Education Beijing China

**Keywords:** automated multiplex serology, cancer, chronic infection, prospective study, relative risk

## Abstract

Several oncogenic pathogens cause specific cancers, but uncertainties remain about many other chronic infections, combined pathogen effects and evidence from non‐European populations. We conducted a case‐cohort study of ~30,000 site‐specific incident cancer cases and > 8000 subcohort participants nested within the China Kadoorie Biobank. Baseline plasma was assayed for IgG antibodies against 47 antigens from 20 pathogens (16 viruses, 3 bacteria, 1 parasite) using an Automated Multiplex Serology assay. We described seroprevalence by age, sex, areas and lifestyle factors; estimated adjusted odd ratios for correlates of pathogen seropositivity in the subcohort using multivariable logistic regression and adjusted hazard ratios for overall and selected cancers using Prentice‐weighted Cox models. Among subcohort participants, seroprevalence for most pathogens varied and was significantly associated with sex, region and birth cohort. Participants were seropositive for a mean of ~10 pathogens. Compared with seronegative participants, those seropositive for seven pathogens had significantly higher overall cancer risk, particularly for HCV (HR = 2.18, 95% CI: 1.90–2.49), CMV (1.23, 1.08–1.40) and HSV‐2 (1.14, 1.09–1.18) and HPV‐16 oncogenes (e.g., E6: 1.57, 1.40–1.75). Lower risks were observed for HSV‐1 (0.88, 0.81–0.95) and among those with fewer co‐infections. There were expected positive associations of liver cancer with HBV (2.29, 2.06–2.54) and HCV (7.05, 4.31–11.54) and of stomach cancer with *H. pylori* (1.91, 1.68–2.17). In Chinese adults, multiple chronic infections were associated with risk of overall and certain selected cancers. Further research is warranted to investigate pathogen‐specific and co‐infection‐related risks of site‐specific cancers.

## Abstract (highlights)

What's new?

While several oncogenic pathogens cause site‐specific cancers, uncertainties remain about many other chronic infections and combined pathogen effects, especially in non‐Western populations. Using a large case–cohort study nested within the China Kadoorie Biobank, the authors found that co‐infection was common, with a mean of 10 pathogens per individual. Compared with seronegative participants, those seropositive for seven pathogens, particularly HCV, CMV, HSV‐2 and HPV‐16, had a higher overall cancer risk. Lower risks were observed for participants exhibiting HSV‐1 seropositivity or with fewer co‐infections. These findings indicate that multi‐pathogen exposure is prevalent and relevant to subsequent cancer risk in Chinese adults.

## Graphical abstract

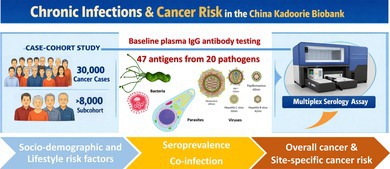

**Abbreviations**
BKV: BK virus

*C. burnetii*

:
*Coxiella burnetii*

CI: confidence interval
CKB: China Kadoorie Biobank
CMV: Cytomegalovirus

*C. trachomatis*

:
*Chlamydia trachomatis*

CV: coefficient of variation
DALY: disability‐adjusted life‐year
DKFZ: German Cancer Research Center
EBV: Epstein–Barr virus
GST: glutathione S‐transferase
HBV: hepatitis B virus
HCV: hepatitis C virus
HHV: human herpesviruses
HIV: human immunodeficiency virus
HPV: human papillomavirus

*H. pylori*

:
*Helicobacter pylori*

HR: hazard ratio
HSV: human simplex virus
HTLV: human T‐lymphotropic virus
JCV: JC virus
LOWESS: locally weighted scatterplot smoothing
MCV: Merkel Cell polyomavirus
MFI: median fluorescence intensity
NCD: non‐communicable disease
OR: odd ratio
*T. gondii*
: *Toxoplasma gondii*
VZV: varicella zoster virus

## 1 Introduction

Chronic pathogen infections are estimated to account for > 2 million cancer cases and ~10% of all disability‐adjusted life‐years (DALYs) attributable to non‐communicable diseases (NCDs) worldwide [1, 2]. Several chronic infectious pathogens, such as *Helicobacter pylori* ( *H. pylori* ), Hepatitis B and C viruses (HBV, HCV) and Human Papillomavirus (HPV) have been classified as carcinogenic to humans [3, 4]. Evidence from previous studies has not only informed the development of vaccines but also supported the implementation of community‐based screening programs for certain cancer (e.g., HPV testing for cervical cancer) [5, 6]. Other pathogens (e.g., certain human herpesviruses [HHVs] and polyomaviruses) have also been implicated in cancer pathogenesis, but existing evidence is still limited and less robust [3, 4].

The burden of infectious pathogens and associated disease outcomes varies substantially both between and within populations. These disparities are influenced by a range of factors, including host genetic susceptibility and immune response, demographic and socioeconomic conditions, lifestyle behaviours, environmental exposures and the genetic diversity of pathogens. Moreover, co‐infections with multiple pathogens are common and may significantly affect clinical outcomes [7, 8]. However, most previous sero‐epidemiological studies have chiefly focused on associations of single pathogens with a single or limited number of disease outcomes. Moreover, these studies have included chiefly Western populations, often with small sample sizes or use of retrospective case–control designs. Consequently, important uncertainties remain about the prospective associations between different chronic infections in a wide range of cancers and other NCDs, and how risks may be modified by co‐infection, host immunity, and other lifestyle/environmental factors, many of which differ substantially between different populations.

Because levels of circulating antibodies, particularly IgG, in plasma are relatively stable and easy to measure, serological testing using stored blood samples is commonly employed in infection‐related disease aetiology studies [9]. The advent of high‐throughput Multiplex Serology assays now enables the simultaneous measurement of antibodies against a wide range of pathogens [10]. Using a Luminex bead‐based Automated Multiplex Serology platform, we conducted a case‐cohort study nested within the China Kadoorie Biobank (CKB) to comprehensively investigate the aetiological roles of chronic infections with multiple pathogens in the development of various diseases, with a particular focus on site‐specific cancers. This paper provides an overview of the study design and presents findings on the seroprevalence, correlates, and co‐infection patterns of multiple pathogens in a large population of middle‐aged and older Chinese adults. Additionally, we examined the associations between multiple chronic infections and the risk of overall cancer incidence and replicated certain well‐established infection‐cancer relationships.

## 2 Methods

### 2.1 Study Population and Data Collection

Details of the CKB design, study population and survey methods have been described previously [11]. Briefly, the CKB recruited 512,724 adults (59% women) aged 30–79 years from 10 geographically diverse regions across China. At baseline, participants completed an interviewer‐administered, laptop‐based questionnaire covering sociodemographic, lifestyle factors (e.g., smoking, alcohol, diet consumption) and personal and family medical history. In addition, trained technicians performed a range of physical measurements (e.g., anthropometry, blood pressure). Periodic resurveys were conducted every 4–5 years in a randomly selected 5% subset of surviving participants.

At both baseline and resurveys, a 10 mL non‐fasting venous blood sample was collected from each participant into an EDTA vacutainer. Samples were maintained at < 4°C and transported to the regional laboratory for centrifugation and aliquoting into three plasma cryovials and one DNA‐containing buffy coat cryovial within 24 h of collection. The cryovials were then stored in a −40°C freezer for 3–4 months before being couriered on dry ice to the central blood repository in Beijing for storage at −80°C. Two frozen plasma aliquots per participant were subsequently transported to Oxford for storage in liquid nitrogen vapour.

### 2.2 Follow Up for Cancer Incidence

The vital status and health of study participant were monitored through electronic linkage with established morbidity and mortality registries, as well as the national health insurance system which provides near‐universal coverage in the study regions [11]. Reported disease events were coded according to the International Classification of Disease 10th Revision (ICD‐10), by trained staff who were blinded to baseline information. By 1.1.2019, 33,977 had developed incident cancer and < 1% were lost‐to‐follow‐up since baseline. All data analysis used CKB data release 19.03.

### 2.3 Case‐Cohort Sero‐Epidemiological Study

**FIGURE 1:**
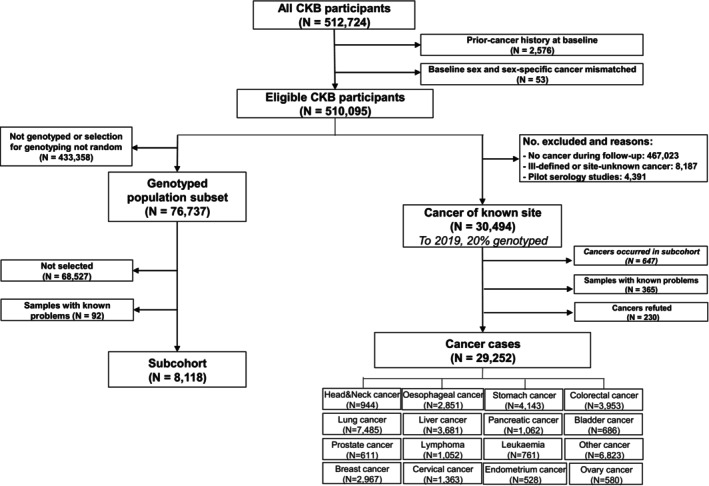
Flow diagram of study design and sample selection in CKB. Selection for the subcohort used random sampling selection. Individuals may be included in more than one study arm; CKB, China Kadoorie Biobank.

We conducted a case‐cohort sero‐epidemiological study nested within the CKB cohort. The study includes a subcohort of individuals randomly selected from a ‘modified baseline representative subcohort’ with genotyping data available and no history of cancer at baseline as well as all participants who developed incident various site‐specific cancers during follow‐up to January 1st 2019 (Figure 1). Overall, 37,370 individuals (29,252 cancer cases and 8118 subcohort participants) were selected, among whom 311 were also included in previous pilot sero‐epidemiological studies [12, 13] and there were 631 subcohort participants who also developed cancers during the follow‐up. A 40 μL subaliquot of baseline plasma from each participant was transferred to a 96‐well plate in a randomised order to avoid batch effects, blinded to case status. The plates of samples were shipped on dry ice from the Wolfson laboratory in Oxford to the German Cancer Research Center (DKFZ) in Heidelberg for the serology assay measurements.

### 2.4 Automated Multiplex Serology Assay

In collaboration with DKFZ, we developed a custom‐designed Multiplex Serology antigen panel tailored for the Chinese population, modelled on validated pathogens developed for the UKB study [14]. Based on findings from two pilot studies in CKB [13, 15], and assay optimizations, the final CKB multiplex serology panel comprised 47 antigens from 20 infectious pathogens that are putatively associated with cancers and other NCDs, and most had been externally validated for measurement in large‐scale prospective studies.

A Luminex bead‐based Automated Multiplex Serology platform has been used to simultaneously measures IgG antibody levels against multiple pathogen‐specific antigens using 10 μL of plasma. The assay is based on a glutathione S‐transferase (GST) capture ELISA combined with fluorescent‐bead technology, with extensive validation procedures applied [10, 14, 16, 17, 18, 19, 20]. For each antigen, the assay generates standardized median fluorescence intensity (MFI) values based on measurements from at least 100 beads per sample. Seropositivity for each specific antigen was defined by DKFZ using cut‐off values derived from validating available pathogen‐specific serological assays for HSV‐1, HSV‐2, VZV, EBV (EBNA1, EA‐D, VCAp18, ZEBRA), CMV, HBV, HCV (NS3), *C. trachomatis* , *T. gondii* and *C. burnetii* on the Automated Multiplex Serology platform, or using percentile‐based distribution plots from general population samples for HHV‐6/7, HPV, polyomaviruses, HIV‐1, HTLV‐1 and HCV (Core). For *H. pylori* antigens a finite mixture model was used that defines the cut‐off as minimum between seropositive and seronegative distributions. The cut‐offs for the NPC‐specific EBV antigens BFRF1, BGLF2 and BXLF1 were determined using the definition of 95% specificity among controls of a previous NPC case‐cohort study [13]. Overall seropositivity for each pathogen presented with two or more antigens was defined using pre‐specified combinations of antigen seropositivity (Table S1).

Detailed descriptions of assay procedures and quality control measures are available elsewhere [21]. Overall, the median specificity was 99%, median sensitivity was 93% and median coefficients of variation (CVs) based on antibody levels from identical samples ranged between 2.8% and 11.7% across all antigens, with no detectable batch effects over a six‐month sample measurement period. Reproducibility was also evaluated using 91 inter‐batch duplicate samples, with correlation coefficients of individual antigen ranging from 0.815 (HPV18‐L1) to 0.998 (BKV) (Table S2).

### 2.5 Statistical Analysis

Among subcohort participants, seroprevalence of each pathogen was calculated separately, overall and by sex, year of birth and study area. We also estimated the pairwise Spearman correlation coefficients between antigen‐ and pathogen‐specific seropositivity. To visualize the temporal trends, weighted scatterplots with locally weighted scatterplot smoothing (LOWESS) curves were used to plot pathogen‐specific seroprevalence by birth year. We calculated the total number of pathogens for which each participant was seropositive at baseline for overall participants and among subgroups defined by major baseline characteristics. The distribution and patterns of seroprevalence were also explored in cancer cases.

Among subcohort participants, cross‐sectional associations between pathogen seropositivity and various sociodemographic, lifestyle, and health‐related factors were assessed using multivariable logistic regression models. These models were adjusted for age, sex, and study region and reported odds ratios (ORs) with 95% confidence intervals (CIs) for each potential correlate studied.

To assess prospective associations between seropositivity for each pathogen and antigen with subsequent risk of cancer incidence, Cox proportional hazards models were fitted with the Prentice pseudo‐partial likelihood method, appropriate for the case‐cohort design [22], adjusting for age, sex, study region, education, smoking, alcohol, BMI and family history of cancer. Participants were followed from baseline until the earliest of any cancer diagnosis (or diagnosis of specific cancer type studied), death, loss‐to‐follow‐up, or 1st January 2019. Adjusted hazard ratios (HRs) and 95% CIs were estimated for cancer incidence according to seropositivity status for each pathogen/antigen. The proportional hazards assumption was assessed using scaled Schoenfeld residual plots and corresponding chi‐square tests. A correction for multiple testing was performed using both the Benjamini‐Hochberg false discovery rate (FDR) and Bonferroni methods, where appropriate.

Extremely prevalent or rare infections with seroprevalence ≤ 0.1% or ≥ 99.9% were excluded from the association analyses. All statistical analyses were performed using R version 4.5.0.

## 3 Results

### 3.1 Baseline Characteristics of Study Participants

Among 8118 subcohort participants, the mean (SD) age was 52.0 (10.7) years and 58.4% were women. Similar patterns for most baseline characteristics were observed between the subcohort and the entire CKB cohort, although the subcohort included slightly more urban residents, individuals with a prior history of CVD or diabetes, and fewer highly educated participants. Compared to the subcohort, the cancer cases tend to be older and were more likely to be men, regular smokers or alcohol drinkers (in both men and women), and to report poorer health and a prior history of blood transfusion, medical conditions such as cirrhosis/hepatitis, and family history of cancer (Table 1).

**TABLE 1:** Baseline characteristics of CKB participants in multiplex infection cancer sub‐study.

Characteristics	Cancer cases	Subcohort	All CKB participants
	(*N* = 29,252)	(*N* = 8118)	(*N* = 512,724)
*Socio‐demographic, lifestyle, physical measures*			
Age, years, Mean (SD)	57.2 (10.2)	52.0 (10.7)	51.5 (10.6)
Women, %	52.7	58.4	59.0
Urban resident, %	49.1	48.1	44.1
Education > 6 years, %	43.2	43.9	49.2
Annual household income < 20,000 (Yuan), %	42.5	40.1	42.7
Ever regular smoker, %			
Men	79.9	74.1	74.4
Women	5.4	4.5	3.2
Ever regular alcohol drinker, %			
Men	52.4	46.7	48.2
Women	4.7	4.2	4.3
Physical activity, MET‐h/day, Mean (SD)	18.5 (13.2)	19.1 (14.2)	21.1 (13.5)
BMI, kg/m^2^, Mean (SD)	23.7 (3.5)	23.7 (3.5)	23.7 (3.5)
Waist circumference, cm, Mean (SD)	80.9 (10.1)	80.4 (9.9)	80.3 (10.1)
SBP, mmHg, Mean (SD)	133.9 (21.9)	134.6 (21.6)	131.1 (21.8)
RPG, mmol/L, Mean (SD)	6.3 (2.6)	6.2 (2.4)	6.1 (2.6)
HBsAg+, %	5.4	2.7	3.1
Blood transfusion, %	5.4	4.8	4.3
*Prior diseases reported at baseline, %*			
CHD or Stroke	6.4	6.7	4.5
Diabetes	4.3	4.4	3.2
Cirrhosis/Hepatitis	2.0	1.3	1.2
Emphysema/Bronchitis	3.7	3.5	2.6
Tuberculosis	2.1	2.1	1.5
Peptic ulcer	4.8	4.3	3.9
Poor self‐rated health	11.7	10.8	10.4
Family history of cancer, %	19.0	17.4	16.6

*Note:* Adjusted for age (10‐year age groups), sex and region (10 regions) where appropriate.

Abbreviations: BMI, Body Mass Index; CHD, Chronic Heart Disease; MET, Metabolic Equivalent of Task; RPG, Random Plasma Glucose; SBP, Systolic Blood Pressure.

### 3.2 Seroprevalence, Co‐Infection and Correlates of Pathogens

The distribution of MFI values for antibodies against each antigen and the corresponding antigen seropositivity levels are shown in Figure S1 and Table S3, respectively. In the subcohort, the seroprevalence of the 20 studied pathogens ranged from 0.1% (HIV‐1 and HTLV‐1) to 99.9% (EBV) among Chinese adults. The most prevalent infections included the herpesviruses (except for HSV‐2), polyomaviruses, HBV and *H. pylori* , all with seroprevalence exceeding 50% (Table 2, Figure S2). Significant variations in seroprevalence by sex, region and birth cohort were observed for most pathogens. The seroprevalence of eight pathogens (i.e., HSV‐1, HSV‐2, CMV, HHV‐6, HHV‐7, BKV, HPV‐18, *C. trachomatis* ) was significantly higher in women, while VZV, HBV, JCV were more prevalent in men (Table 2). Across the 10 study regions, comparisons of the highest vs. lowest seroprevalence revealed at least 2‐fold differences for HSV‐2, HBV, HPV‐16, HPV‐18, *C. trachomatis, T. gondii* and *H. pylori* (Figure S3). Decreasing temporal trends in seroprevalence across birth cohorts were observed for HSV‐1, HBV, JCV, MCV, HPV‐18 and *C. trachomatis* , while an increasing trend was found for HHV‐6, HHV‐7 and *T. gondii* (Table 2), with similar trends in seroprevalence were observed further by individual birth year (Figure S4).

**TABLE 2:** Seroprevalence for infectious pathogens in subcohort, by sex, area and birth cohort, % (SE).

Pathogen	Sex		Area		Birth cohort			All (*n* = 8118)
	Male (*n* = 3283)	Female (*n* = 4835)	Rural (*n* = 4286)	Urban (*n* = 3832)	< 1950 (*n* = 2914)	1950–1959 (*n* = 2554)	> 1959 (*n* = 2650)	
*Human Herpes Virus*								
HSV‐1	**97.1 (0.29)**	**98.4 (0.18)**	**97.1 (0.26)**	**98.8 (0.18)**	**98.4 (0.23)**	**98.3 (0.25)**	**96.9 (0.34)**	97.9 (0.16)
HSV‐2	**7.4 (0.46)**	**9.3 (0.42)**	**9.3 (0.44)**	**7.7 (0.43)**	9.1 (0.53)	7.5 (0.52)	8.9 (0.55)	8.5 (0.31)
VZV	**92.4 (0.46)**	**88.2 (0.46)**	**91.4 (0.43)**	**88.2 (0.52)**	90.0 (0.55)	90.5 (0.58)	89.1 (0.61)	89.9 (0.33)
EBV	99.8 (0.07)	99.9 (0.04)	99.9 (0.06)	99.9 (0.05)	99.9 (0.06)	99.8 (0.08)	99.9 (0.07)	99.9 (0.04)
CMV	**98.2 (0.23)**	**99.6 (0.09)**	99.1 (0.15)	99.0 (0.16)	99.1 (0.17)	99.0 (0.20)	99.0 (0.20)	99.0 (0.11)
HHV‐6	**53.0 (0.87)**	**62.6 (0.70)**	**56.5 (0.76)**	**61.1 (0.79)**	**55.3 (0.92)**	**60.5 (0.97)**	**60.7 (0.95)**	58.7 (0.55)
HHV‐7	**81.6 (0.68)**	**89.7 (0.44)**	**85.5 (0.54)**	**87.4 (0.54)**	**83.6 (0.69)**	**87.4 (0.66)**	**88.5 (0.62)**	86.4 (0.38)
*Hepatitis virus*								
HBV	**63.0 (0.84)**	**55.5 (0.71)**	**56.8 (0.76)**	**60.4 (0.79)**	**65.3 (0.88)**	**57.4 (0.98)**	**52.0 (0.97)**	58.5 (0.55)
HCV	0.5 (0.13)	0.2 (0.07)	**0.5 (0.11)**	**0.2 (0.07)**	0.2 (0.08)	0.5 (0.14)	0.4 (0.12)	0.4 (0.07)
*Human Retrovirus*								
HTLV‐1	0.1 (0.04)	0.1 (0.04)	0.1 (0.05)	0.1 (0.04)	0.1 (0.07)	0.0 (0.04)	0.0 (0.04)	0.1 (0.03)
HIV‐1	0.1 (0.04)	0.1 (0.05)	0.0 (0.03)	0.1 (0.06)	0.1 (0.06)	0.0 (0.00)	0.2 (0.08)	0.1 (0.03)
*Human Polyomavirus*								
BKV	**89.9 (0.52)**	**92.1 (0.39)**	**90.2 (0.45)**	**92.5 (0.43)**	90.9 (0.53)	92.0 (0.54)	90.9 (0.56)	91.3 (0.31)
JCV	**71.1 (0.79)**	**65.0 (0.69)**	**65.6 (0.73)**	**69.6 (0.74)**	**75.3 (0.80)**	**64.7 (0.95)**	**61.6 (0.95)**	67.5 (0.52)
MCV	57.3 (0.86)	55.3 (0.72)	**53.8 (0.76)**	**58.8 (0.80)**	**60.2 (0.91)**	**56.6 (0.98)**	**51.3 (0.97)**	56.1 (0.55)
*Human Papillomavirus*								
HPV‐16	5.5 (0.40)	6.1 (0.34)	5.6 (0.35)	6.1 (0.39)	6.5 (0.46)	5.5 (0.45)	5.5 (0.44)	5.8 (0.26)
HPV‐18	**4.5 (0.36)**	**5.9 (0.34)**	**4.7 (0.32)**	**6.0 (0.38)**	**6.9 (0.47)**	**4.8 (0.42)**	**4.2 (0.39)**	5.3 (0.25)
*Bacteria and Parasite*								
*C. trachomatis*	**41.1 (0.86)**	**46.7 (0.72)**	**42.5 (0.76)**	**46.6 (0.81)**	**54.9 (0.92)**	**42.6 (0.98)**	**34.8 (0.93)**	44.4 (0.55)
*H. pylori*	70.1 (0.80)	68.4 (0.67)	**61.4 (0.74)**	**77.7 (0.67)**	**69.7 (0.85)**	**71.1 (0.90)**	**66.6 (0.92)**	69.1 (0.51)
*C. burnetii*	11.3 (0.55)	11.0 (0.45)	10.9 (0.48)	11.3 (0.51)	11.1 (0.58)	11.6 (0.63)	10.6 (0.60)	11.1 (0.35)
*T. gondii*	23.5 (0.74)	23.3 (0.61)	**24.9 (0.66)**	**21.7 (0.67)**	**22.2 (0.77)**	**22.7 (0.83)**	**25.3 (0.84)**	23.4 (0.47)

*Note:* Bold values denote statistical significance at the *p* < 0.05 level.

Abbreviations: BKV, BK polyomavirus; *C. burnetii* , *Coxiella burnetii* ; *C. trachomatis* , *Chlamydia trachomatis* ; CMV, Cytomegalovirus; EBV, Epstein–Barr virus; *H. pylori* , *Helicobacter pylori* ; HBV, hepatitis B virus; HCV, hepatitis C virus; HHV, human herpes virus; HIV, human immunodeficiency virus; HPV, human papillomavirus; HSV, human simplex virus; HTLV, human T‐lymphotropic virus; JCV, JC polyomavirus; MCV, Merkel Cell polyomavirus; *T. gondii*, *Toxoplasma gondii*; VZV, varicella zoster virus.

Co‐infection was prevalent among study participants. The mean number of seropositive pathogens was 9.7 (SD 1.9) in subcohort participants and 31.3% participants were seropositive for > 10 pathogens. However, a declining trend in co‐infection by birth‐cohort was observed, with the proportion decreasing from 40.8% among people born before 1940 to 26.3% in those born after 1960 (Figure S5). The mean numbers of coinfected pathogens were slightly higher in women, urban residents, less educated participants, and overweight/obese participants compared to their counterparts (Table S4).

Correlations between pathogens were generally low. However, a moderate correlation was observed between HPV‐16 and HPV‐18 (*r* = 0.57) (Figure S6). Stronger correlations were evident among antigens from the same pathogen, for example, for HBV, *r* = 0.91 between HBc and HBe, and for *H. pylori* , for example, *r* = 0.44 between CagA and GroEL (Figure S7).

### 3.3 Cross‐Sectional Associations Between Demographic and Lifestyle Factors and Pathogen Seropositivity

Several factors demonstrated significant associations with at least four pathogens seropositivity, including sex, age, birth cohort, area, smoking, alcohol drinking, poor self‐reported health status/health conditions (Table 3). For example, women had over four‐fold higher risk of seropositivity for CMV (OR = 4.23, 95% CI: 2.56–6.99) and nearly two‐fold higher risk for HSV‐1 (1.88, 1.38–2.56) and HHV‐7 (1.92, 1.69–2.19) while with ~30%–40% lower risk of seropositivity for VZV (0.62, 0.53–0.73) and HBV (0.74, 0.67–0.81), compared to men. And, as expected, HBV seropositivity was strongly associated with baseline HBsAg positivity (12.13, 7.02–20.98) and *H. pylori* seropositivity with a prior history of peptic ulcer (1.37, 1.04–1.80).

**TABLE 3:** Adjusted ORs of seropositivity for selected pathogens by baseline characteristic, among subcohort.

	Demographic and socio‐economic factors				Lifestyle factors				Medical history		
	Age (per year increase)	Women	Urban	Education > 6 years	Regular smoker (men)	Regular alcohol drinker (men)	Overweight (BMI > 25 kg/m^2^)	Fresh fruit	HBsAg+	Poor self‐rated health	Family history of cancer
HSV‐1	**1.03 (1.01, 1.04)**	**1.88 (1.38, 2.56)**	**2.35 (1.67, 3.29)**	0.99 (0.68, 1.43)	0.66 (0.39, 1.12)	0.97 (0.62, 1.50)	1.30 (0.90, 1.87)	1.43 (0.82, 2.49)	**0.44 (0.22, 0.88)**	0.69 (0.42, 1.12)	**0.63 (0.42, 0.95)**
HSV‐2	1.01 (1.00, 1.01)	**1.31 (1.11, 1.55)**	**0.80 (0.68, 0.94)**	1.02 (0.84, 1.24)	1.22 (0.89, 1.67)	1.08 (0.82, 1.44)	1.01 (0.84, 1.20)	0.99 (0.78, 1.26)	1.33 (0.85, 2.07)	1.12 (0.86, 1.46)	0.96 (0.76, 1.22)
VZV	1.00 (1.00, 1.01)	**0.62 (0.53, 0.73)**	**0.70 (0.61, 0.81)**	0.99 (0.83, 1.19)	**1.57 (1.19, 2.07)**	1.21 (0.92, 1.59)	0.86 (0.74, 1.01)	1.00 (0.83, 1.22)	1.06 (0.68, 1.64)	0.82 (0.65, 1.03)	1.02 (0.84, 1.24)
CMV	1.01 (0.99, 1.03)	**4.23 (2.56, 6.99)**	0.85 (0.55, 1.33)	**0.56 (0.32, 0.99)**	**3.80 (2.22, 6.51)**	1.53 (0.87, 2.69)	1.06 (0.64, 1.75)	0.91 (0.48, 1.71)	2.69 (0.37, 19.55)	0.90 (0.43, 1.91)	0.76 (0.42, 1.38)
HHV‐6	**0.99 (0.99, 0.99)**	**1.46 (1.34, 1.60)**	**1.22 (1.12, 1.34)**	**0.82 (0.73, 0.92)**	1.03 (0.88, 1.21)	**0.85 (0.73, 0.98)**	0.99 (0.89, 1.09)	**0.86 (0.76, 0.98)**	1.01 (0.77, 1.33)	1.09 (0.94, 1.27)	1.02 (0.90, 1.15)
HHV‐7	**0.98 (0.98, 0.99)**	**1.92 (1.69, 2.19)**	**1.20 (1.05, 1.36)**	1.01 (0.86, 1.19)	**1.28 (1.05, 1.57)**	1.00 (0.83, 1.20)	0.87 (0.76, 1.00)	1.01 (0.84, 1.22)	**1.95 (1.18, 3.23)**	0.88 (0.71, 1.08)	1.02 (0.86, 1.21)
HBV	**1.02 (1.02, 1.03)**	**0.74 (0.67, 0.81)**	**1.13 (1.03, 1.24)**	0.93 (0.83, 1.05)	1.04 (0.87, 1.23)	0.92 (0.79, 1.08)	0.98 (0.89, 1.08)	0.89 (0.78, 1.01)	**12.13 (7.02, 20.98)**	**1.20 (1.03, 1.41)**	1.04 (0.92, 1.18)
HCV	0.97 (0.94, 1.01)	0.48 (0.23, 1.00)	0.45 (0.20, 1.01)	0.82 (0.34, 2.01)	2.09 (0.59, 7.41)	1.83 (0.67, 5.01)	0.76 (0.33, 1.75)	1.62 (0.49, 5.37)	0.00 (0.00, 100.00)	2.20 (0.87, 5.51)	0.58 (0.20, 1.72)
BKV	1.00 (0.99, 1.01)	**1.31 (1.12, 1.54)**	**1.34 (1.14, 1.56)**	**0.78 (0.65, 0.95)**	**0.71 (0.53, 0.96)**	0.92 (0.72, 1.17)	1.13 (0.95, 1.34)	0.93 (0.75, 1.17)	0.90 (0.57, 1.42)	0.95 (0.74, 1.24)	1.01 (0.81, 1.25)
JCV	**1.03 (1.02, 1.03)**	**0.77 (0.70, 0.85)**	**1.16 (1.06, 1.28)**	1.05 (0.93, 1.18)	0.94 (0.79, 1.12)	1.04 (0.89, 1.23)	1.00 (0.90, 1.11)	0.92 (0.80, 1.05)	1.05 (0.79, 1.39)	**1.28 (1.08, 1.52)**	**1.16 (1.02, 1.32)**
MCV	**1.01 (1.01, 1.02)**	0.93 (0.85, 1.02)	**1.20 (1.10, 1.31)**	1.01 (0.90, 1.13)	1.06 (0.90, 1.24)	0.93 (0.80, 1.08)	**1.11 (1.00, 1.22)**	1.09 (0.96, 1.23)	0.92 (0.70, 1.20)	1.16 (0.99, 1.35)	1.11 (0.98, 1.26)
HPV‐16	1.00 (0.99, 1.01)	1.13 (0.93, 1.36)	1.10 (0.91, 1.32)	0.91 (0.72, 1.15)	0.82 (0.59, 1.15)	0.93 (0.68, 1.28)	1.13 (0.92, 1.38)	0.82 (0.62, 1.07)	0.85 (0.47, 1.54)	1.09 (0.79, 1.49)	0.94 (0.72, 1.23)
HPV‐18	**1.02 (1.01, 1.03)**	**1.38 (1.12, 1.70)**	**1.25 (1.03, 1.51)**	0.81 (0.63, 1.03)	0.80 (0.55, 1.15)	0.92 (0.64, 1.30)	1.11 (0.90, 1.37)	0.81 (0.62, 1.07)	0.54 (0.25, 1.16)	1.07 (0.78, 1.48)	0.93 (0.71, 1.23)
*C. trachomatis*	**1.04 (1.03, 1.04)**	**1.35 (1.23, 1.48)**	**1.13 (1.03, 1.23)**	1.01 (0.90, 1.13)	**1.21 (1.03, 1.44)**	**1.21 (1.04, 1.41)**	1.09 (0.99, 1.21)	**1.20 (1.06, 1.36)**	1.18 (0.90, 1.55)	0.95 (0.82, 1.11)	1.08 (0.96, 1.22)
*H. pylori*	**1.01 (1.00, 1.01)**	**0.90 (0.82, 1.00)**	**2.18 (1.97, 2.40)**	1.02 (0.90, 1.16)	1.16 (0.97, 1.40)	1.01 (0.85, 1.19)	1.09 (0.98, 1.22)	0.92 (0.80, 1.06)	1.31 (0.96, 1.79)	0.87 (0.74, 1.02)	1.09 (0.95, 1.25)
*C. burnetii*	1.00 (0.99, 1.01)	0.96 (0.84, 1.11)	1.04 (0.91, 1.20)	1.12 (0.94, 1.33)	0.91 (0.71, 1.17)	**1.27 (1.01, 1.59)**	1.02 (0.88, 1.19)	0.88 (0.72, 1.07)	0.65 (0.40, 1.05)	0.97 (0.76, 1.24)	0.94 (0.77, 1.14)
*T. gondii*	**0.99 (0.99, 1.00)**	0.98 (0.88, 1.09)	**0.85 (0.76, 0.94)**	0.94 (0.82, 1.07)	**1.22 (1.01, 1.49)**	1.05 (0.88, 1.25)	**1.13 (1.01, 1.27)**	0.86 (0.74, 1.01)	0.86 (0.63, 1.19)	**0.81 (0.68, 0.98)**	1.05 (0.91, 1.21)

*Note:* ORs: odds ratios; Adjusted for age (10‐year age groups), sex and region (10 regions) where appropriate; Compared Yes versus No groups for each specific demographic and socio‐economic, lifestyle factor and medical history; Bold values denote statistical significance at the *p* < 0.05 level.

Abbreviation: ORs, odds ratios.

### 3.4 Prospective Associations Between Chronic Infections and Cancer Risks

Compared with the subcohort, cancer cases had significantly higher seroprevalence of several pathogens, including HBV (63.7% in cancer cases vs. 58.5% in subcohort individuals), HCV (0.7% vs. 0.4%), JCV (71.2% vs. 67.5%) and *C. trachomatis* (49.3% vs. 44.4%) (Figure S2). The mean number of seropositive pathogens was slightly higher among cancer cases (9.9 [1.8]), and 34.7% participants were seropositive for > 10 pathogens, which declined across birth cohorts from 40.7% (< 1940) to 27.4% (≥ 1960) (Figure S5). In addition to factors observed in the subcohort, a history of blood transfusion and, among men, non‐regular alcohol consumption were associated with a higher co‐infection burden in cancer cases (Table S4). Nevertheless, seroprevalence patterns by sex, region, and birth cohort for most pathogens were similar between cases and subcohort, and pathogen‐ and antigen‐specific correlation patterns largely mirrored those in the subcohort (Table S5, Figures S6–S9).

**FIGURE 2:**
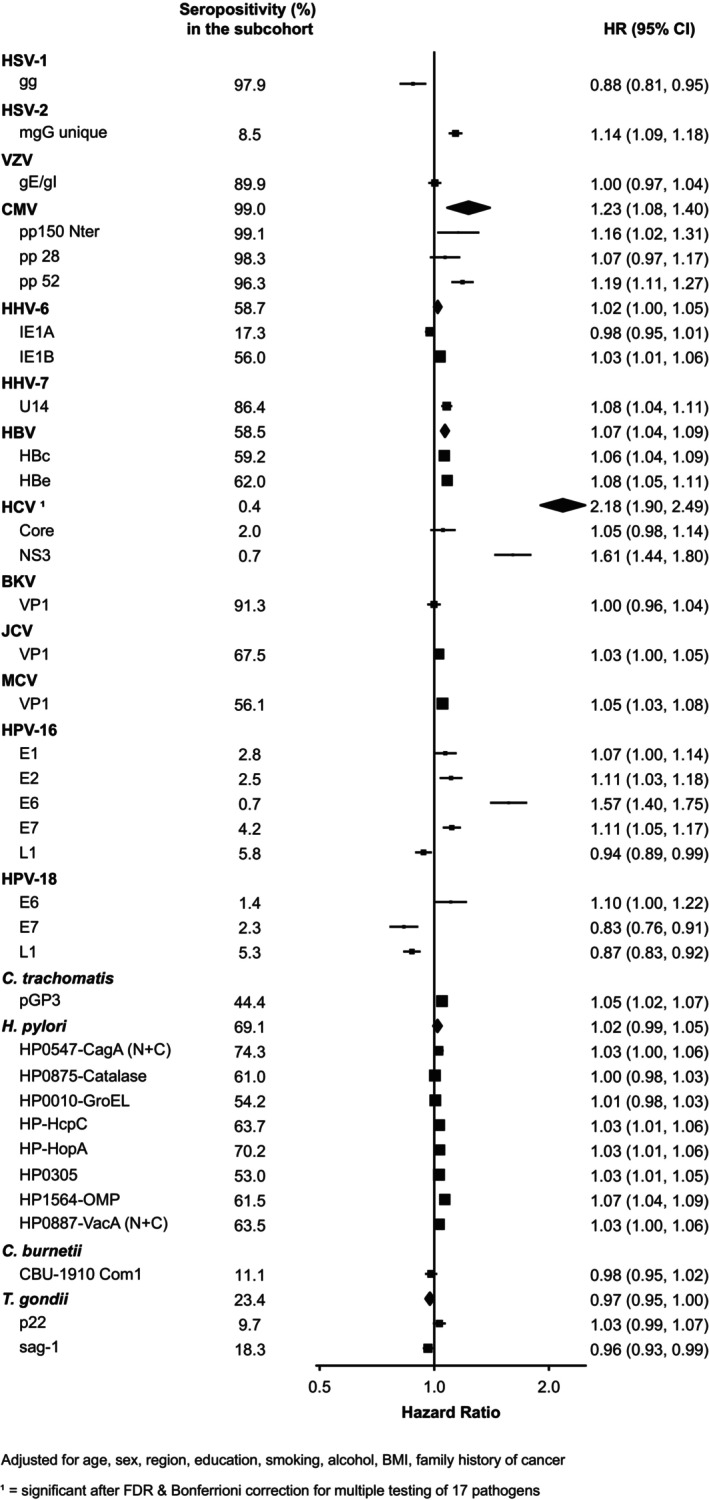
Adjusted HRs for overall cancer incidence from multiple pathogens in Chinese adults. Cox proportional hazards models were fitted with the Prentice pseudo‐partial likelihood method, appropriate for the case‐cohort design. Models were adjusted for age, sex, study region, education, smoking, alcohol drinking, BMI and the family history of cancer, with time since recruitment as the underlying timescale. The diamonds represent adjusted HRs at pathogen level (if ≥ 2 antigens involved to define the pathogen seropositivity status) and the squares represent adjusted HRs at antigen level, both with the area inversely proportional to the variance of the logHRs, and the horizontal lines represent their corresponding 95% CIs. BKV, BK virus; *C. burnetii* , *Coxiella burnetii* ; *C. trachomatis* , *Chlamydia trachomatis* ; CMV, Cytomegalovirus; EBV, Epstein–Barr virus; *H. pylori* , *Helicobacter pylori* ; HBV, Hepatitis B virus; HCV, Hepatitis C virus; HHV, Human herpes virus; HIV, Human immunodeficiency virus; HPV, Human papillomavirus; HSV, Human simplex virus; HTLV, Human T‐lymphotropic virus; JCV, JC virus; MCV, Merkel Cell polyomavirus; *T. gondii*, *Toxoplasma gondii*; VZV, Varicella zoster virus.

After adjustment for sociodemographic, lifestyle factors and family history of cancer, overall cancer incidence was positively associated with seropositivity to HSV‐2 (1.14, 1.09–1.18), CMV (1.23, 1.08–1.40), HHV‐7 (1.08, 1.04–1.11), HBV (1.07, 1.04–1.09), HCV (2.18, 1.90–2.49), JCV (1.03, 1.00–1.05), MCV (1.05, 1.03–1.08) and *C. trachomatis* (1.05, 1.02–1.07) compared with their seronegative counterparts. Only HCV remain statistically significant after either FDA or Bonferoni correction for multiple testing. Antigen‐level analyses showed similar patterns where multiple antigens were available. In contrast, an inverse association was observed for seropositivity to HSV‐1 (0.88, 0.81–0.95). For HPV‐16 and HPV‐18, overall cancer risk was significantly elevated for the E6 oncoprotein (HPV‐16 E6: 1.57, 1.40–1.75 and HPV‐18 E6: 1.10, 1.00–1.22) whereas inverse associations were found for L1 of both types and for HPV‐18 E7 (Figure 2). Compared with participants coinfected with 10 pathogens, those with fewer co‐infections had a significantly lower risk of overall cancer, especially ≤ 6 pathogens (0.53, 0.44–0.63). Conversely, individuals with 11 co‐infections had an elevated risk (1.11, 1.08–1.14), but with no further increase observed at higher co‐infection burdens (Figure S10).

**FIGURE 3:**
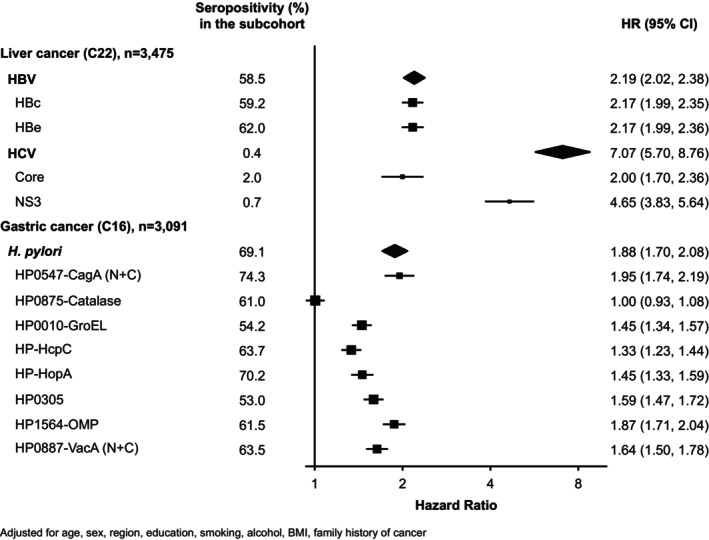
Adjusted HRs for liver cancer from HBV and HCV and for gastric cancers from *H. pylori* in Chinese adults. HBV, Hepatitis B virus; HCV, Hepatitis C virus; *H. pylori* , *Helicobacter pylori* . Conventions as in Figure 2.

We further confirmed the well‐established associations for risk of liver cancer from seropositivity of HBV (HR = 2.19, 95% CI: 2.02–2.38) and HCV (7.07, 5.07–8.76), and for risk of gastric cancer from *H. pylori* seropositivity (1.88, 1.70–2.08). Except Catalase of *H. pylori* , all measured individual antigens of these three pathogens were also associated with these cancers (Figure 3). For gastric cancer subsites, replicating the results from our pilot study [23], after excluded the first 2 years' follow‐up (to avoid the reverse causality due to decreased *H. pylori* antibody level from gastric atrophy), *H. pylori* infection was associated with increased risk for both cardia (2.19, 1.43–3.35) and non‐cardia gastric cancer (3.13, 2.39–4.10). The increased risk was consistently showed for most *H. pylori* antigens measured (Figure S11).

## 4 Discussion

In this large case‐cohort sero‐epidemiological study among Chinese adults, we observed substantial variation in the seroprevalence of multiple infectious pathogens, by sex, birth cohort and geographic region. Seropositivity to several pathogens was associated with a range of sociodemographic, lifestyle, and medical factors, particularly age, sex, region, smoking, alcohol consumption and prior health conditions. While pairwise correlations among the 20 measured pathogens were generally low, co‐infection was common, with a mean of nearly 10 seropositive pathogens among the study population. Seropositivity to several HHVs, hepatitis viruses, MCV, *C. trachomatis* and the HPV E6 oncoprotein was associated with increased overall cancer risk, whereas seropositivity to HSV‐1 and fewer pathogen co‐infections were associated with reduced risk. Except for HCV, these results should be interpreted with caution and regarded as exploratory following stringent correction for multiple testing. Consistent with prior evidence, we further confirmed well‐established associations of HBV and HCV with liver cancer, and of *H. pylori* with gastric cancer.

The seroprevalence estimates for major infections observed in this study were broadly consistent with findings from previous nationwide and regional surveys or meta‐analyses in China [24, 25, 26, 27, 28, 29, 30, 31, 32, 33, 34, 35, 36, 37]. However, few studies have systematically examined the sero‐epidemiology of multiple chronic infections in general populations. Our seroprevalence estimates of viral infections of HBV, HCV, HHVs and HPyVs were generally consistent with those from a pooled case–control study using a similar Multiplex Serology assay among 214 controls from three population‐based prospective cohorts in Shanghai and Singapore [38]. Estimates from our previous pilot study in 2000 CKB subcohort also aligned closely with the present study results [15], except for a lower seroprevalence of *T. gondii* in the current analysis due to improved *T. gondii* antigen peptide design for the more recent assay [18]. Notably, pilot studies in both CKB and UKB (10,000 UKB participants) have revealed marked differences in the seroprevalence of certain pathogens across populations [14, 15].

The observed associations between key demographic factors, such as age, sex and region, and the seroprevalence of many pathogens were consistent with previous findings [15, 29]. Moreover, the present study further identified several lifestyle factors (e.g., smoking, alcohol drinking) and self‐reported poor health and prior disease also associate with seropositivity for most pathogens studied. These results support the need to consider such factors as potential confounders in infection‐disease association analyses and suggest they may also be relevant targets for infection prevention strategies.

The replicated associations of HBV and HCV with liver cancer and *H. pylori* with gastric cancer serve as positive controls and validate the robustness of the Multiplex Serology data in this large‐scale epidemiological study. Unlike previous studies that primarily used HBsAg to identify active HBV infection which often provide higher magnitude of risk estimates, our study used antibodies against HBc and HBe, which indicates host immune response to the virus past or ongoing infection. The associations with gastric cancer from *H. pylori* infection were consistent with findings from our previous pilot case‐cohort study, in which we included 500 each of cardia and non‐cardia gastric cancer within the CKB [23].

To our knowledge, this is the first large‐scale study to assess the association between multiple pathogens, individually and combined, and risk of overall cancer incidence. The observed associations with overall cancer risk should, however, be considered hypothesis generating, as they may reflect both a cumulative infection burden and a relatively high proportion of infection‐related cancers. Future studies are warranted to investigate pathogen‐ and antigen‐ and co‐infection‐specific associations with site‐specific cancers, while accounting for potential reverse causality from specific infections, rather than suggesting a generalized carcinogenic effect across all cancer types.

Strengths of this study include the prospective study design, large numbers of participants recruited from diverse regions within China, comprehensive sociodemographic, lifestyle, medical history data collected at baseline plus long‐term follow‐up for all hospitalized events and the use of a high‐throughput, validated, Automated Multiplex Serology platform. This assay has demonstrated strong reliability in previous studies, including pilot work in both CKB and UKB, with robust reproducibility and low rates of sero‐conversion and sero‐reversion in repeat samples measured at different sample collection points [12, 13, 14, 15, 23, 39, 40, 41]. Moreover, participants in CKB were generally not covered by vaccination programmes in China for any of the pathogens studied, including HBV and HPV [42]. Using this rich dataset, we have identified key potential confounders to inform future analyses of infection–disease associations. These analyses will support the evaluation of both putative and novel infectious contributors to cancer and other NCDs.

However, several limitations exist. First, the Multiplex Serology assay is an epidemiological screening tool designed to detect cumulative exposure. Second, although this is the largest study of its kind, statistical power to detect associations may be limited for pathogens with very high or very low prevalence (e.g., EBV, HIV‐1, HTLV‐1) in the Chinese population, at least at the pathogen level. Third, the assay does not allow for precise determination of primary infection timing, nor does it capture reactivation or acute infection events. Nevertheless, linkage with health‐care recorded infection diagnoses during the follow‐up may help capture some reactivations and will be incorporated into future analyses. Fourth, the prospective associations between infection pathogens and subsequent cancer risk may reflect participants' immune status or residual confounding by correlated exposures (e.g., aflatoxins in relation to liver cancer). Future research incorporating genetic and multi‐omics data may help elucidate the underlying carcinogenetic mechanisms. Last, we cannot rule out potential effects from regional pathogen eradications efforts, initiatives addressing antibiotic resistance, or nationwide/opportunistic cancer screening programmes in the context of cancer association analyses.

In conclusion, this large case–cohort study nested within the CKB cohort characterised the sero‐epidemiology of multiple chronic infections, identified associations between multiple pathogens and overall cancer risk. The observed relationships with modifiable risk factors highlight the potential for infection control and prevention to reduce the cancer burden in China and elsewhere. Future integration of genomic and other ‐omics data, along with harmonised serological data from other large‐scale cohorts from diverse populations (e.g., UKB, EPIC, CPS‐II), will enable in‐depth investigation into the complex interplay of host–pathogen interactions in cancer and NCD aetiology, and will inform precision public health strategies.

## Author Contributions

**Ling Yang:** conceptualization, data curation, writing – original draft, writing – review and editing, investigation, funding acquisition, methodology, formal analysis, project administration. **Jonathan Clarke:** methodology, formal analysis, writing – review and editing, investigation, visualization. **Lea Kröller:** data curation, writing – review and editing, methodology, validation. **Christiana Kartsonaki:** methodology, formal analysis, writing – review and editing, conceptualization. **Hannah Fry:** project administration, writing – review and editing, resources. **Rima Jeske:** data curation, writing – review and editing, validation. **Andrew Gordon:** project administration, data curation, writing – review and editing. **Sarah Clark:** data curation, project administration, writing – review and editing. **Michael Hill:** data curation, project administration, writing – review and editing. **Daniel Avery:** project administration, writing – review and editing. **Yiping Chen:** data curation, writing – review and editing. **Huaidong Du:** data curation, writing – review and editing. **Jun Lv:** data curation, writing – review and editing. **Dianjianyi Sun:** data curation, writing – review and editing. **Canqing Yu:** data curation, writing – review and editing. **Liming Li:** data curation, writing – review and editing, supervision. **Iona Y. Millwood:** data curation, conceptualization, writing – review and editing, project administration, investigation, methodology. **Tim Waterboer:** conceptualization, investigation, data curation, writing – review and editing, supervision, methodology. **Zhengming Chen:** data curation, conceptualization, funding acquisition, writing – review and editing, supervision.

## Funding

The present study was funded by CRUK Programme Grant (Ref: C16077/A29186). The CKB baseline survey and the first re‐survey were supported by the Kadoorie Charitable Foundation in Hong Kong. The long‐term follow‐up and subsequent resurveys have been supported by Wellcome grants to Oxford University (212946/Z/18/Z, 202922/Z/16/Z, 104085/Z/14/Z, 088158/Z/09/Z) and grants from the National Natural Science Foundation of China (82388102) and the Noncommunicable Chronic Diseases‐National Science and Technology Major Project (2023ZD0510101, 2023ZD0510100). The UK Medical Research Council (MC_UU_00017/1, MC_UU_12026/2, MC_U137686851), Cancer Research UK (C16077/A29186, C500/A16896), and the British Heart Foundation (CH/1996001/9454) provide core funding to the Clinical Trial Service Unit and Epidemiological Studies Unit at Oxford University for the project. The funder of the study had no role in study design, data collection, analysis and interpretation, or in the writing of the report.

## Ethics Statement

The China Kadoorie Biobank (CKB) complies with all required ethical standards for medical research on human subjects. Ethical approvals were obtained and maintained by the relevant institutional ethical research committees in the UK and China.

## Consent

All participants provided written informed consent at baseline, including consent for long‐term storage of biological samples, access to their medical records and the use of anonymised data for future research.

## Conflicts of Interest

The authors declare no conflicts of interest.

## Supporting information


**Table S1:** Pathogens/antigens included in multiplex serology panel and cutoffs.
**Table S2:** Comparison of MFI values of 91 sample that been repeatedly measured twice.
**Figure S1:** Distribution of median fluorescence intensity (MFI) of antigens measured.
**Table S3:** Seropositivity for each antigen measured, by study arm.
**Figure S2:** Seroprevalence (%) of each pathogen by study arm.
**Figure S3:** Seroprevalence (%) of each pathogen in each region, among subcohort.
**Figure S4:** Seroprevalence (%) of each pathogen by year of birth among subcohort.
**Figure S5:** Coinfection of multiple pathogens overall and by birth year, by study arm.
**Table S4:** Mean number of coinfected pathogens by baseline characteristics, by study arm.
**Figure S6:** Spearman's correlation between pathogen seropositivity, by study arm.
**Figure S7:** Spearman's correlation between antigen seropositivity, by study arm.
**Table S5:** Seroprevalence (% (SE)) of each pathogen among incident cancer cases, by sex, region and birth cohort.
**Figure S8:** Seroprevalence (%) for each pathogen in each region, among incident cancer cases.
**Figure S9:** Seroprevalence (%) of each pathogen by year of birth among incident cancer cases.
**Figure S10:** Prospective associations between the number of co‐infected pathogens and risk of overall cancer incidence in Chinese adults.
**Figure S11:** Adjusted HRs for cardia gastric cancer and non‐cardia gastric cancer by *H. pylori*.

## Data Availability

This work has been conducted using the China Kadoorie Biobank (CKB) data release version R19.03, and is based on Research Tracker item number 2025–0018 that includes the serology data. Data access requests should be made to the China Kadoorie Biobank (CKB) Data Access Team [ckbaccess@ndph.ox.ac.uk], and will be reviewed by the CKB Data Access Committee. All source code used for the statistical analyses in this manuscript is publicly available on GitHub (https://github.com/jonathanclarke‐ghub/CKB_infections_overview). Further information is available from the corresponding author upon request.
