# Supplementary material for: Burden and Correlates of Multiple Chronic Infections and Their Associations With Cancer Incidence in Chinese Adults: A Large Case‐Cohort Study
Source: Int J Cancer. 2026 May 18;159(5):1282–95. doi: 10.1002/ijc.70555 (PMC13340953; doi:10.1002/ijc.70555)
Supplement: Supplementary file 1 — Table S1: Pathogens/antigens included in multiplex serology panel and cutoffs. Table S2: Comparison of MFI values of 91 sample that been repeatedly measured twice. Figure S1: Distribution of median fluorescence intensity (MFI) of antigens measured. Table S3: Seropositivity for each antigen measured, by study arm. Figure S2: Seroprevalence (%) of each pathogen by study arm. Figure S3: Seroprevalence (%) of each pathogen in each region, among subcohort. Figure S4: Seroprevalence (%) of each pathogen by year of birth among subcohort. Figure S5: Coinfection of multiple pathogens overall and by birth year, by study arm. Table S4: Mean number of coinfected pathogens by baseline characteristics, by study arm. Figure S6: Spearman's correlation between pathogen seropositivity, by study arm. Figure S7: Spearman's correlation between antigen seropositivity, by study arm. Table S5: Seroprevalence (% (SE)) of each pathogen among incident cancer cases, by sex, region and birth cohort. Figure S8: Seroprevalence (%) for each pathogen in each region, among incident cancer cases. Figure S9: Seroprevalence (%) of each pathogen by year of birth among incident cancer cases. Figure S10: Prospective associations between the number of co‐infected pathogens and risk of overall cancer incidence in Chinese adults. Figure S11: Adjusted HRs for cardia gastric cancer and non‐cardia gastric cancer by H. pylori. [file IJC-159-1282-s001.pdf]

SUPPLEMENTARY APPENDIX

Burden and correlates of multiple chronic infections and their associations with cancer incidence in Chinese adults: a large case-cohort study.

Ling Yang, Jonathan Clarke, Lea Kröller, Christiana Kartsonaki, Hannah Fry, Rima Jeske, Andrew Gordon, Sarah Clark, Michael Hill, Daniel Avery, Yiping Chen, Huaidong Du, Jun Lv, Dianjianyi Sun, Canqing Yu, Liming Li, Iona Y. Millwood, Tim Waterboer, Zhengming Chen

Content List of Supplementary Materials **Page**

Members of the China Kadoorie Biobank collaborative group 2
eTable 1. Pathogens/antigens included in multiplex serology panel and cutoffs 3
eTable 2. Comparison of MFI values of 91 sample that been repeatedly measured twice 4
eFigure 1. Distribution of median fluorescence intensity (MFI) of antigens measured 5
eTable 3. Seropositivity for each antigen measured, by study arm 6
eFigure 2. Seroprevalence (%) of each pathogen by study arm 7
eFigure 3. Seroprevalence (%) of each pathogen in each region, among subcohort 8
eFigure 4. Seroprevalence (%) of each pathogen by year of birth among subcohort 9
eFigure 5. Coinfection of multiple pathogens overall and by birth year, by study arm 10
eTable 4. Mean number of coinfected pathogens by baseline characteristics, by study arm 11
eFigure 6. Spearman's correlation between pathogen seropositivity, by study arm 12
eFigure 7 Spearman's correlation between antigen seropositivity, by study arm 13
eTable 5. Seroprevalence (% (SE)) of each pathogen among incident cancer cases, by sex, region and birth cohort 14
eFigure 8. Seroprevalence (%) for each pathogen in each region, among incident cancer cases 15
eFigure 9. Seroprevalence (%) of each pathogen by year of birth among incident cancer cases 16
eFigure 10. Prospective associations between the number of co-infected pathogens and risk of overall cancer incidence in Chinese adults 17
eFigure 11. Adjusted HRs for cardia gastric cancer and non-cardia gastric cancer by *H. pylori* 18

Members of the China Kadoorie Biobank collaborative group

International Steering Committee: Junshi Chen, Zhengming Chen (PI), Robert Clarke, Rory Collins, Liming Li (PI), Jun Lv, Richard Peto, Robin Walters.

National Co-ordinating Centre, Beijing: Liming Li, Jun Lv, Canqing Yu, Dianjianyi Sun, Yuanjie Pang, Yuting Han, Can Hou, Qingmei Xia, Chao Liu, Pei Pei, Lang Pan, Xiao Han, Honglu Bian, Xinxin Chen.

International Co-ordinating Centre, Oxford: Daniel Avery, Maxim Barnard, Derrick Bennett, Ruth Boxall, Yiping Chen, Zhengming Chen, Jonathan Clarke; Robert Clarke, Huaidong Du, Ahmed Edris Mohamed, Hannah Fry, Prapthi Harish, Pek Kei Im, Andri Iona, Christiana Kartsonaki, Kshitij Kolhe, Hubert Lam, Kuang Lin, James Liu, Iona Millwood, Sam Morris, Qunhua Nie, Alfred Pozarickij, Maryam Rahmati, Paul Ryder, Maruf Sarder, Dan Schmidt, Becky Stevens, Iain Turnbull, Robin Walters, Baihan Wang, Lin Wang, Neil Wright, Ling Yang, Xiaoming Yang, Pang Yao.

10 Regional Co-ordinating Centres:
Qingdao CDC: Zengchang Pang, Ruqin Gao, Kunzheng Lv, Shanpeng Li, Haiping Duan, Shaojie Wang, Yongmei Liu, Ranran Du, Liang Cheng, Xiaocao Tian, Hua Zhang. Licang CDC: Dan Hu, Xiaoyan Zheng, Yujie Wang. Heilongjiang Provincial CDC: Wei Sun, Shichun Yan, Yongzhou. Nangang CDC: Chi Wang, Zhenyuan Wu, Lishun Zhai, Zhaoxi Pang, Shiwen Dong, Li Liu. Hainan Provincial CDC: Dapeng Yin, Bin He, Ying Liu, Xingren Wang, Tingting Ou. Meilan CDC: Xiangyang Zheng, Dewei Zheng, Shuai Yang, Lihui Li, Xingjiao Chen. Jiangsu Provincial CDC: Yan Xu, Jinyi Zhou, Ran Tao, Jian Su, Xikang Fan, Xuejia Chen, Yuxiao Huang. Suzhou CDC: Yan Lu, Yujie Hua, Li Xing, Shuxian Wang, Jianrong Jin, Juping Ma, Jinchao Liu, Kaifei Zhu, Hongfu Ren, Xingfeng Shen. Guangxi Provincial CDC: Ge Zhong, Wei Mao, Zhenzhen Lu, Yanxu Zhong. Liuzhou CDC: Lifang Zhou, Rong Pan, Jian Lan, Xiaoping Tan, Jinxue Tan, Yishan Xie, Liuping Wei, Liyuan Zhou, Sisi Wang. Sichuan Provincial CDC: Xianping Wu, Ningmei Zhang, Xiaofang Chen, Xiaoyu Chang, Zhuo Wang, Yujin He. Pengzhou CDC: Mingqiang Yuan, Ling Wang, Xiaofang Chen, Zhaodong Wang, Qiang Sun, Yang Lin. Gansu Provincial CDC: Faqing Chen, Xiaolan Ren, Lijun Chang, Feiming Zhong. Maiji CDC: Jianjun Feng, Weijie Hu, Xiaofang Zhang, Yalin Chen, Honghong Wang, Jun Wang. Henan Provincial CDC: Linqi Diao, Zhiwei Han, Dengjun Zhu, Kai Kang, Shixian Feng, Wenjie Yang, Huizi Tian, Yali Yan, Bing Han, Li Gao, Shaofang Li, Tianfang Xing, Wei Tang. Huixian CDC: Xiaolin Li, Huarong Sun, Xiaocong Zhao, Ying Li, Chen Hu, Pan He, Xukui Zhang, Yuanyuan Jin, Lan Luo. Zhejiang Provincial CDC: Min Yu, Ruying Hu, Hao Wang, Weiwei Gong, Jieming Zhong, Meng Wang, Chunxiao Xu, Keqing Gong. Tongxiang CDC: Hao Xu, Yuan Cao, Kaixu Xie, Lingli Chen, Xiaomei Tu, Junlong Pan. Hunan Provincial CDC: Xiaojun Li, Li Yin, Huilin Liu, Yuan Liu, Lei Yin, Xian Xie, Jing Wang. Liuyang CDC: Bo Xiao, Zongwei Deng, Yuan Peng, Libo Zhang, Chan Qu, li Deng,Qili Jiang, Yanling Chen.

eTable 1. Pathogens/antigens included in multiplex serology panel and cutoffs

Pathogen	Antigen	Antigen seropositive cutoff (MFI)	Algorithm for pathogen seropositivity
**Human Herpes Virus**			
HSV-1	gG	458	above cutoff
HSV-2	mgGunique	1578	above cutoff
VZV	gE/gI	400	above cutoff
EBV	BFRF1	2658	-
	BGLF2	4666	-
	BXLF1	6755	-
	EA-D	799	≥ 2 out of 4 above cutoff
	EBNA1	922	
	VCAp18	4085	
	ZEBRA	296	
CMV	pp150 N	820	≥ 2 out of 3 above cutoff
	pp28	551	
	pp52	4633	
HHV-6	IE1A	2000	≥ 1 out of 2 above cutoff
	IE1B	1200	
HHV-7	U14	120	above cutoff
**Hepatitis virus**			
HBV	HBc	518	2 out of 2 above cutoff
	HBe	556	
HCV	Core	1000	2 out of 2 above cutoff
	NS3	2658	
**Human Retrovirus**			
HIV-1	env	700	2 out of 2 above cutoff
	gag	2000	
HTLV-1	env	2000	2 out of 2 above cutoff
	gag	750	
**Human Polyomavirus**			
BKV	VP1	1000	above cutoff
JCV	VP1	800	above cutoff
MCV	VP1	700	above cutoff
**Human Papillomavirus**			
HPV-16	E1	900	-
	E2	600	-
	E6	1000	-
	E7	700	-
	L1	500	above cutoff
HPV-18	E6	800	-
	E7	700	-
	L1	500	above cutoff
**Bacteria and Parasite**			
C. trachomatis	pGP3	2708	above cutoff
H. Pylori	CagA (N)	994	≥ 4 out of 8 above cutoff
	Catalase	260	
	GroEL	870	
	HcpC	185	
	HopA	155	
	HP0305	148	
	HP1564	841	
	VacA (C)c	260	
C. Burnetti	Com1	171	above cutoff
T. gondii	p22trunc	147	2 out of 2 above cutoff
	sag-1D1	312	

MFI: Median Fluorescence Intensities; - : cancer-related antigen measured which was not included in 'Algorithm for pathogen seropositivity'

eTable 2. Comparison of MFI values of 91 sample that been repeatedly measured twice

Pathogen	Antigen	Spearman's rank correlation coefficient (ρ)
**Human Herpes Virus**		
HSV-1	gG	0.984
HSV-2	mgGunique	0.974
VZV	gE/gI	0.992
EBV	BFRF1	0.989
	BGLF2	0.996
	BXLF1	0.995
	EA-D	0.996
	EBNA1	0.988
	VCAp18	0.995
	ZEBRA	0.993
CMV	pp150 N	0.994
	pp28	0.996
	pp52	0.994
HHV-6	IE1A	0.988
	IE1B	0.995
HHV-7	U14	0.988
**Hepatitis virus**		
HBV	HBc	0.993
	HBe	0.996
HCV	Core	0.900
	NS3	0.968
**Human Retrovirus**		
HIV-1	env	0.924
	gag	0.939
HTLV-1	env	0.986
	gag	0.901
**Human Polyomavirus**		
BKV	VP1	0.998
JCV	VP1	0.997
MCV	VP1	0.989
**Human Papillomavirus**		
HPV-16	E1	0.953
	E2	0.926
	E6	0.877
	E7	0.953
	L1	0.921
HPV-18	E6	0.856
	E7	0.873
	L1	0.815
**Bacteria and Parasite**		
C. trachomatis	pGP3	0.981
H. pylori	CagA (N)	0.997
	Catalase	0.990
	GroEL	0.997
	HcpC	0.968
	HopA	0.986
	HP0305	0.980
	HP1564	0.997
	VacA (C)c	0.993
C. burnetti	Com1	0.894
T. gondii	p22trunc	0.887
	sag-1D1	0.960

eFigure 1. Distribution of median fluorescence intensity (MFI) of antigens measured

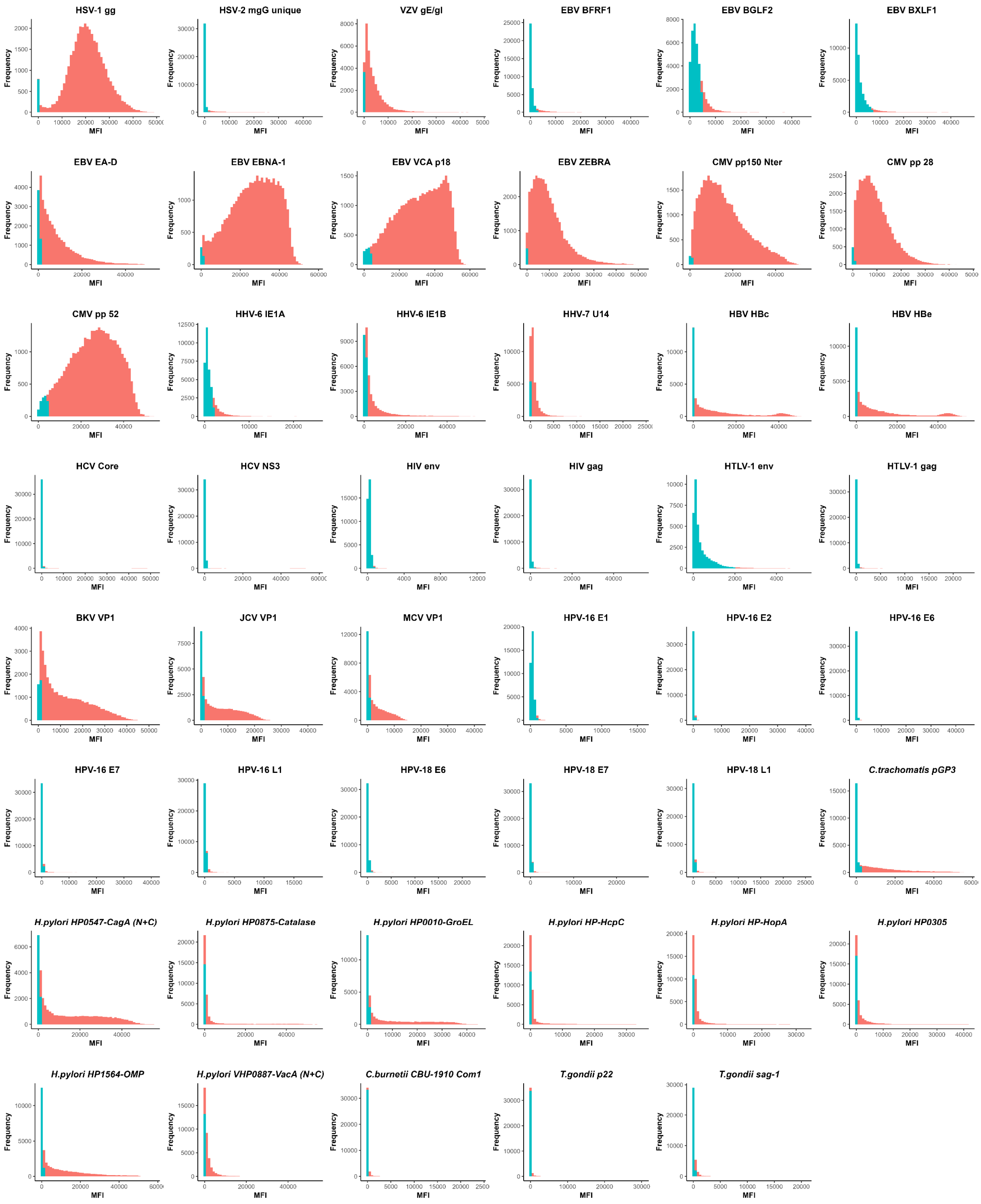

For each antigen, orange and blue represent sero-status of positive and negative, respectively.

eTable 3. Seropositivity for each antigen measured, by study arm

Pathogen	Antigen	Seropositivity	
		Subcohort	Cancer cases
**Human Herpes Virus**			
HSV-1	gG	97.9	97.9
HSV-2	mgGunique	8.5	8.9
VZV	gE/gl	89.9	90.4
EBV	BFRF1	8.5	9.5
	BGLF2	14.4	18.2
	BXLF1	6.0	7.4
	EA-D	85.8	86.2
	EBNA1	98.8	98.9
	VCAp18	97.4	97.4
	ZEBRA	98.6	98.8
CMV	pp150 N	99.1	99.2
	pp28	98.3	98.4
	pp52	96.3	97.0
HHV-6	IE1A	17.3	17.4
	IE1B	56.0	54.7
HHV-7	U14	86.4	85.6
**Hepatitis virus**			
HBV	HBc	59.2	64.4
	HBe	62.0	67.2
HCV	Core	2.0	2.3
	NS3	0.7	1.1
**Human Retrovirus**			
HIV-1	env	1.1	1.3
	gag	2.0	2.1
HTLV-1	env	2.1	2.3
	gag	1.6	1.8
**Human Polyomavirus**			
BKV	VP1	91.3	91.1
JCV	VP1	67.5	71.2
MCV	VP1	56.1	58.8
**Human Papillomavirus**			
HPV-16	E1	2.8	2.9
	E2	2.5	2.9
	E6	0.7	1.0
	E7	4.2	4.6
	L1	5.8	5.5
HPV-18	E6	1.4	1.4
	E7	2.3	1.8
	L1	5.3	5.1
**Bacteria and Parasite**			
C. trachomatis	pGP3	44.4	49.3
H. pylori	CagA (N)	74.3	76.3
	Catalase	61.0	61.2
	GroEL	54.2	56.4
	HcpC	63.7	64.6
	HopA	70.2	71.5
	HP0305	53.0	54.9
	HP1564	61.5	63.5
	VacA (C)c	63.5	65.1
C. burnetti	Com1	11.1	11.0
T. gondii	p22trunc	9.7	9.9
	sag-1D1	18.3	16.9

eFigure 2. Seroprevalence (%) of each pathogen by study arm

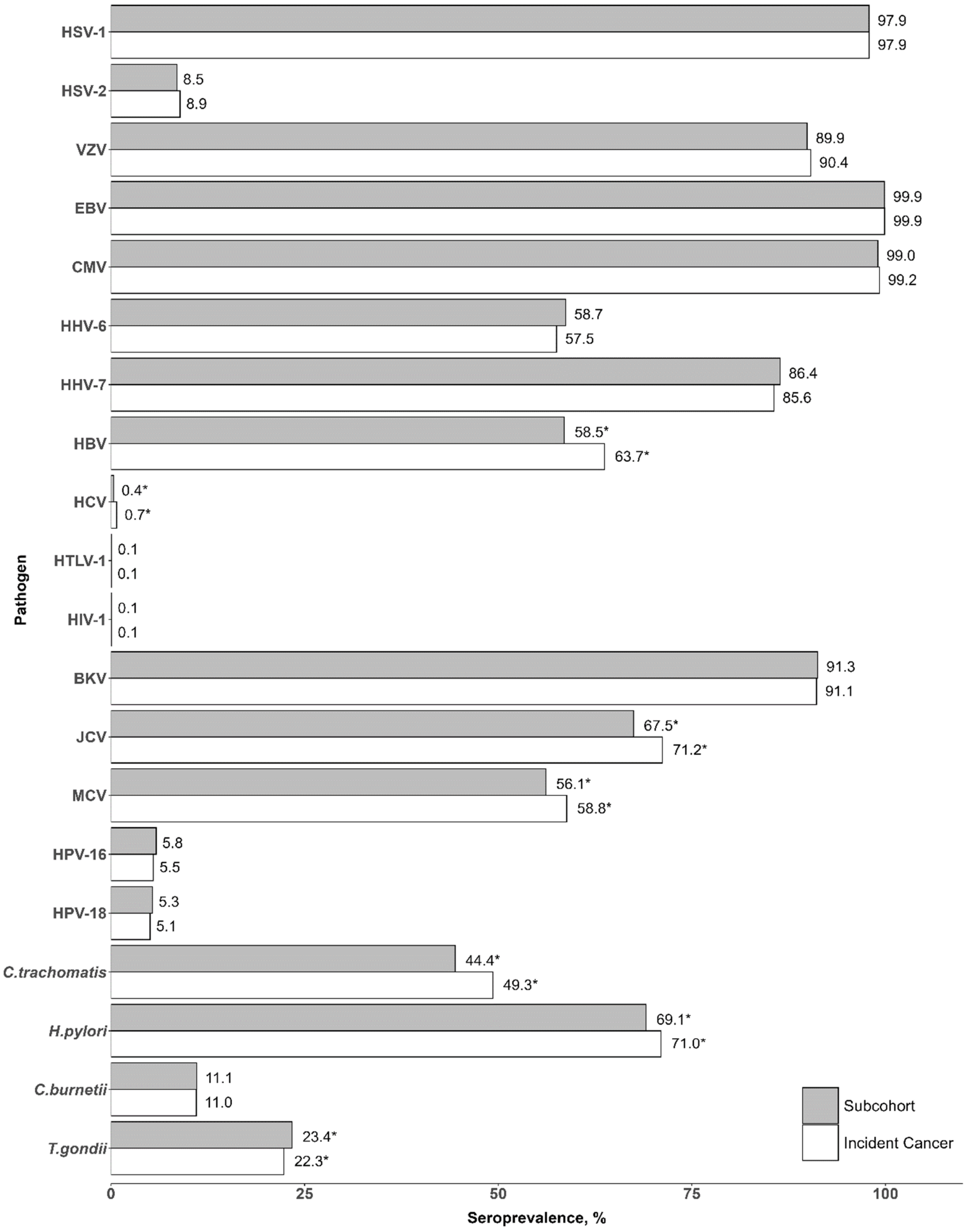

The grey bar represents Subcohort participants and the white bar represents Incident cancer cases.

eFigure 3. Seroprevalence (%) of each pathogen in each region, among subcohort

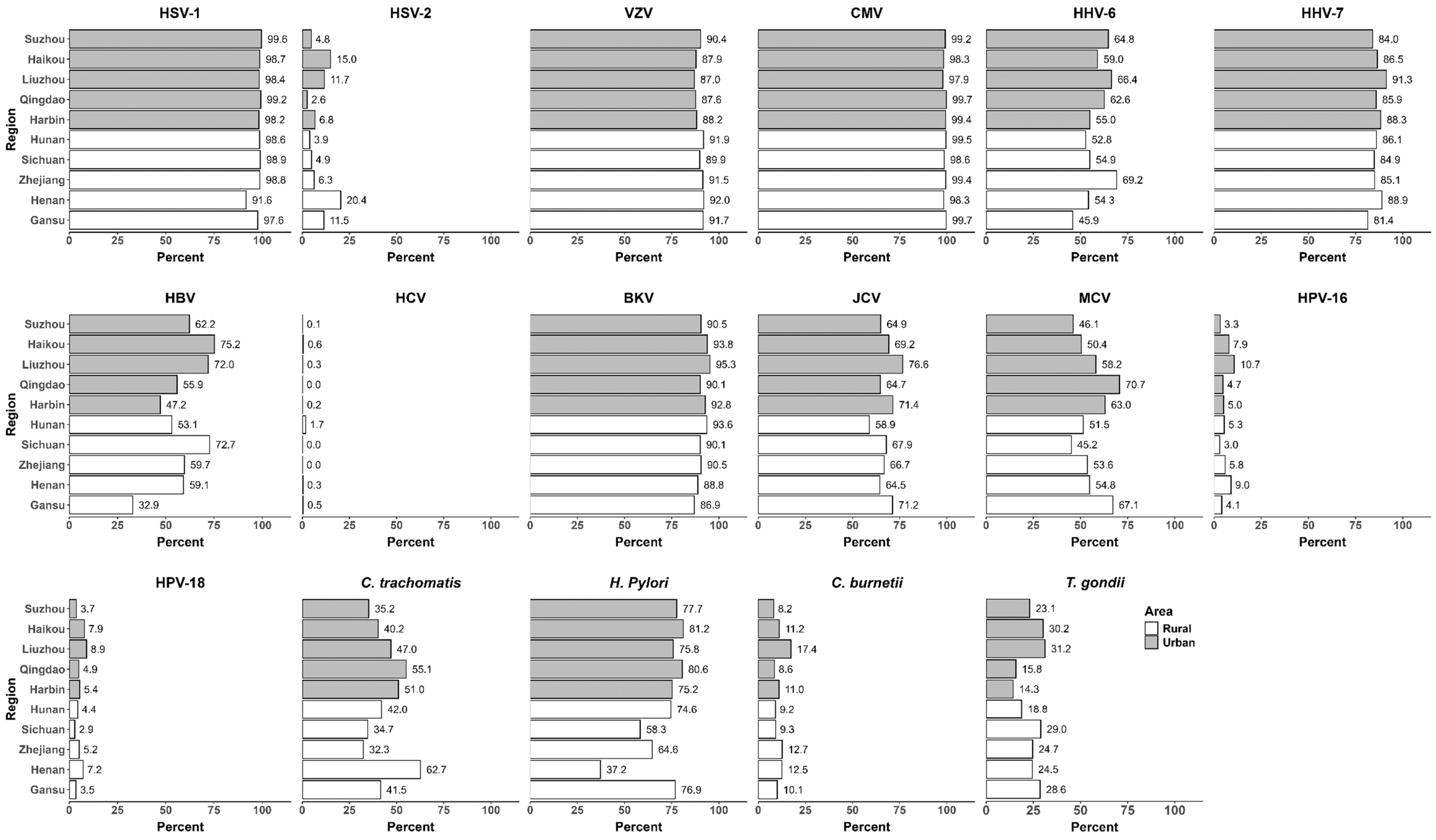

The grey bar represents urban participants and the white bar represents rural participants.

eFigure 4. Seroprevalence (%) of each pathogen by year of birth among subcohort

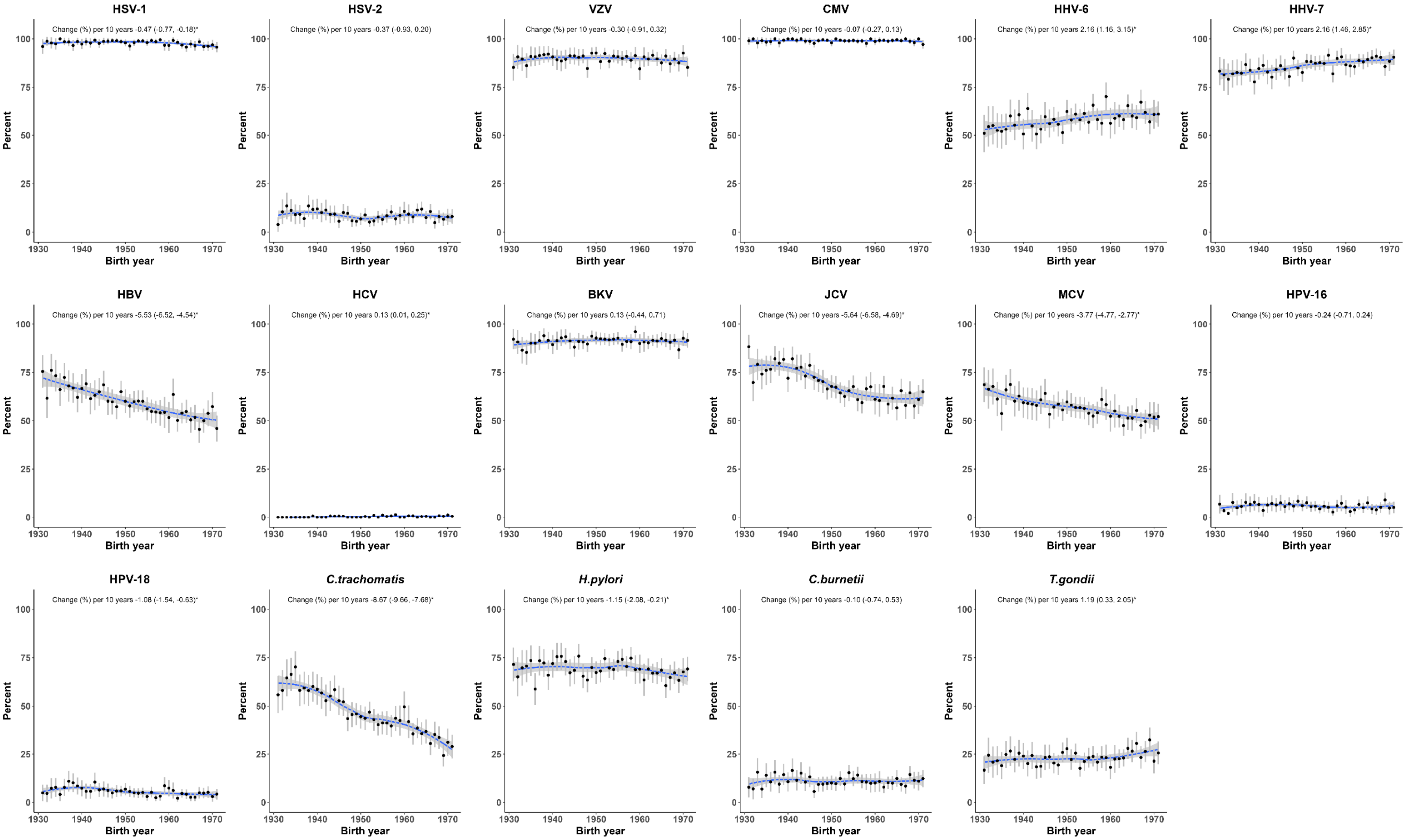

Dots and grey lines represent sero-prevalence estimates and 95% CI for every single birth year. The blue line represents estimates for these sero-prevalences derived from LOESS method, and the shaded areas show the 95% CI. Due to few participants that were born before 1932 or after 1971, those who were born between 1927 and 1931, or 1971 and 1976 were combined, separately.
LOESS, locally weighted scatterplot smoothing

eFigure 5. Coinfection of multiple pathogens overall and by birth year, by study arm

Subcohort

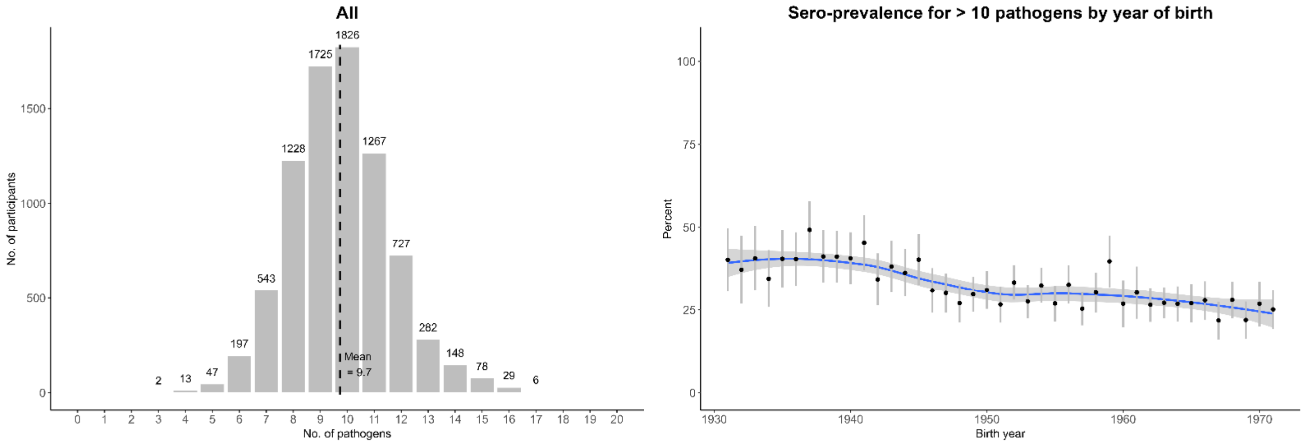

Incident Cancer

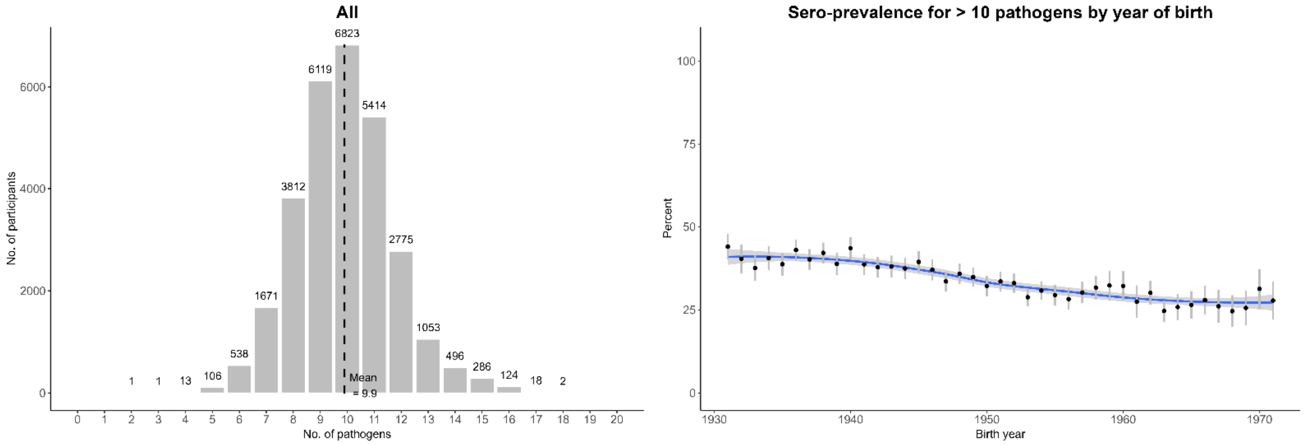

The left panels show the number of seropositive infections in the subcohort participants (upper panel), and incident cancer cases (lower panel). The dashed lines indicate the mean number of seropositive pathogens among study participants. The two right panels show the seroprevalence of >10 pathogens by year of birth within each study arm. Conventions are as in eFigure 4.

eTable 4. Mean number of coinfected pathogens by baseline characteristics, by study arm

Baseline characteristic		Subcohort	Cancer cases
Age (years)	< 40	**9.40 (1.85)**	**9.49 (1.81)**
	40 to 49	**9.58 (1.86)**	**9.61 (1.81)**
	50 to 59	**9.74 (1.83)**	**9.83 (1.79)**
	60 to 69	**10.10 (1.89)**	**10.09 (1.77)**
	70 +	**10.08 (1.81)**	**10.15 (1.76)**
Sex	Women	**9.77 (1.86)**	**9.92 (1.81)**
	Men	**9.67 (1.88)**	**9.86 (1.78)**
Area	Urban	**9.93 (1.88)**	**10.02 (1.80)**
	Rural	**9.56 (1.84)**	**9.77 (1.78)**
Schooling	<= 6 years	**9.78 (1.84)**	**9.93 (1.79)**
	> 6 years	**9.69 (1.88)**	**9.85 (1.81)**
Ever regular smoker (men)	Yes	9.69 (1.85)	9.85 (1.78)
	No	9.61 (1.95)	9.89 (1.79)
Ever regular alcohol drinker (men)	Yes	9.63 (1.86)	**9.82 (1.75)**
	No	9.71 (1.89)	**9.90 (1.81)**
BMI, kg/m²	Normal, or below	**9.69 (1.87)**	**9.88 (1.81)**
	Overweight	**9.79 (1.84)**	**9.91 (1.77)**
	Obese	**9.95 (1.92)**	**10.02 (1.83)**
Blood transfusion	Yes	9.86 (1.90)	**10.03 (1.82)**
	No	9.73 (1.86)	**9.89 (1.80)**
HBsAg+	Yes	**10.12 (1.63)**	**10.19 (1.63)**
	No	**9.72 (1.87)**	**9.88 (1.81)**
CHD or Stroke	Yes	**10.05 (1.81)**	**10.16 (1.81)**
	No	**9.72 (1.87)**	**9.87 (1.79)**
Diabetes	Yes	9.84 (1.84)	9.95 (1.74)
	No	9.73 (1.87)	9.89 (1.80)
Cirrhosis/Hepatitis	Yes	9.97 (1.93)	**10.11 (1.65)**
	No	9.73 (1.86)	**9.89 (1.80)**
Emphysema/Bronchitis	Yes	9.72 (1.76)	9.85 (1.83)
	No	9.73 (1.87)	9.90 (1.80)
Tuberculosis	Yes	9.92 (1.99)	**10.06 (1.73)**
	No	9.73 (1.86)	**9.89 (1.80)**
Peptic Ulcer	Yes	9.89 (1.81)	**10.01 (1.83)**
	No	9.73 (1.87)	**9.89 (1.80)**
Self-rated health	Poor	9.77 (1.90)	9.89 (1.76)
	Fair, good or excellent	9.73 (1.86)	9.89 (1.80)
Family history of cancer	Yes	9.78 (1.81)	9.86 (1.72)
	No	9.72 (1.87)	9.90 (1.81)

Bold values denote statistically significant difference at the $p<0.05$ level.

eFigure 6. Spearman's correlation between pathogen seropositivity, by study arm

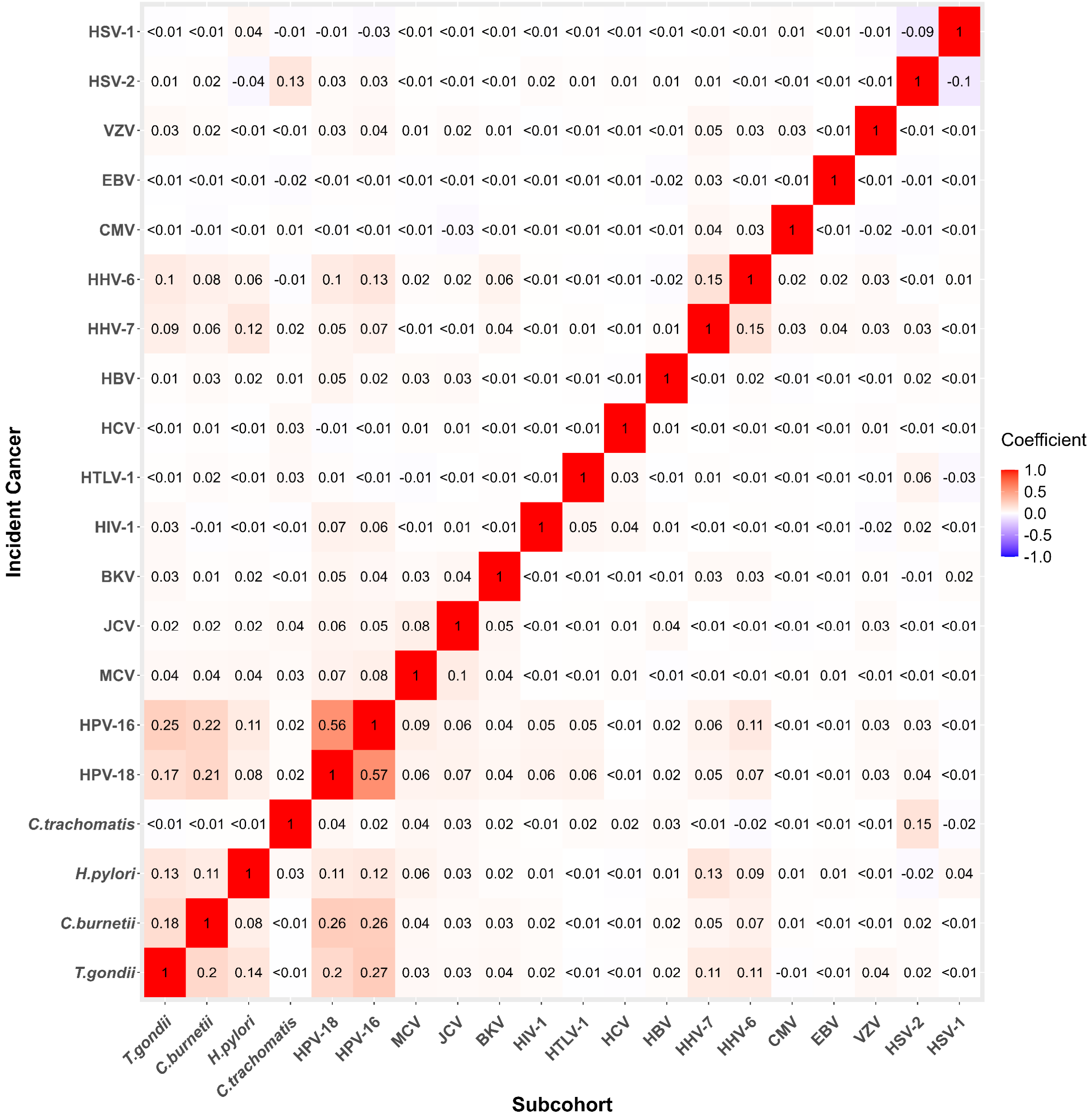

The upper-left matrix shows the Spearman's correlations between pathogens among incident cancer cases, while the lower-right matrix shows the correlations between pathogens among subcohort participants.

eFigure 7 Spearman's correlation between antigen seropositivity, by study arm

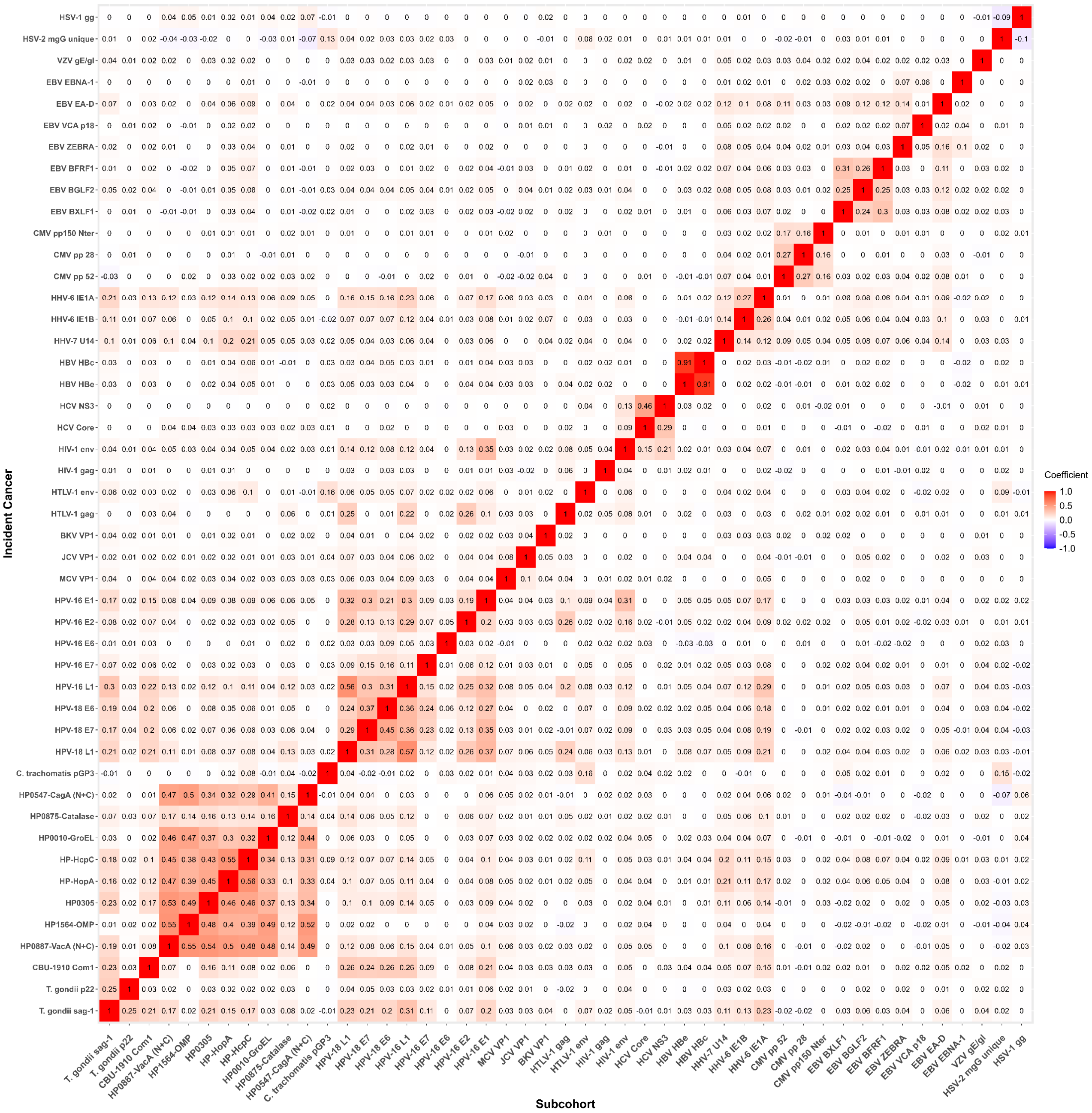

The upper-left matrix shows the Spearman's correlations between antigens among incident cancer cases, while the lower-right matrix shows the correlations between antigens among subcohort participants.

eTable 5. Seroprevalence (% (SE)) of each pathogen among incident cancer cases, by sex, region and birth cohort

	Sex		Area		Birth cohort			
**Pathogen**	**Male (n=14,133)**	**Female (n=15,119)**	**Rural (n=14,876)**	**Urban (n=14,376)**	**< 1950 (n=16,148)**	**1950-1959 (n=8,370)**	**> 1959 (n=4,734)**	**All (n=29,252)**
**Human Herpes Virus**								
HSV-1	**97.3 (0.14)**	**98.5 (0.10)**	**97.1 (0.14)**	**98.7 (0.10)**	**98.2 (0.11)**	**98.1 (0.15)**	**96.6 (0.26)**	97.9 (0.08)
HSV-2	**8.0 (0.23)**	**9.8 (0.24)**	**9.9 (0.24)**	**8.0 (0.23)**	9.1 (0.23)	8.2 (0.30)	9.7 (0.43)	8.9 (0.17)
VZV	**92.6 (0.22)**	**88.2 (0.26)**	**91.6 (0.23)**	**89.0 (0.26)**	91.1 (0.22)	90.3 (0.32)	88.0 (0.47)	90.4 (0.17)
EBV	99.8 (0.04)	99.9 (0.02)	99.8 (0.03)	99.9 (0.02)	99.9 (0.03)	99.8 (0.04)	100.0 (0.00)	99.9 (0.02)
CMV	**98.9 (0.09)**	**99.6 (0.05)**	99.4 (0.06)	99.1 (0.08)	99.3 (0.07)	99.3 (0.09)	98.8 (0.16)	99.2 (0.05)
HHV-6	**51.0 (0.42)**	**63.7 (0.39)**	**55.0 (0.41)**	**60.1 (0.41)**	**55.7 (0.39)**	**58.6 (0.54)**	**61.8 (0.71)**	57.5 (0.29)
HHV-7	**81.6 (0.33)**	**89.3 (0.25)**	**84.5 (0.30)**	**86.8 (0.28)**	**83.8 (0.29)**	**87.1 (0.37)**	**89.3 (0.45)**	85.6 (0.21)
**Hepatitis virus**								
HBV	**69.1 (0.39)**	**58.7 (0.40)**	**63.6 (0.39)**	**63.8 (0.40)**	**68.2 (0.37)**	**60.8 (0.53)**	**53.5 (0.72)**	63.7 (0.28)
HCV	0.8 (0.07)	0.7 (0.07)	**0.9 (0.08)**	**0.6 (0.06)**	0.6 (0.06)	0.8 (0.10)	1.0 (0.14)	0.7 (0.05)
**Human Retrovirus**								
HTLV-1	0.0 (0.02)	0.1 (0.02)	0.0 (0.02)	0.1 (0.02)	0.0 (0.02)	0.1 (0.03)	0.1 (0.04)	0.1 (0.01)
HIV-1	0.1 (0.02)	0.1 (0.02)	0.1 (0.02)	0.1 (0.02)	0.1 (0.02)	0.1 (0.03)	0.1 (0.04)	0.1 (0.02)
**Human Polyomavirus**								
BKV	**89.8 (0.25)**	**92.3 (0.22)**	**90.7 (0.24)**	**91.5 (0.23)**	90.8 (0.23)	91.5 (0.31)	91.4 (0.41)	91.1 (0.17)
JCV	**75.4 (0.36)**	**67.2 (0.38)**	**71.2 (0.37)**	**71.1 (0.38)**	**76.8 (0.33)**	**66.3 (0.52)**	**60.9 (0.71)**	71.2 (0.26)
MCV	60.6 (0.41)	57.2 (0.40)	**56.8 (0.41)**	**60.9 (0.41)**	**61.8 (0.38)**	**56.9 (0.54)**	**52.2 (0.73)**	58.8 (0.29)
**Human Papillomavirus**								
HPV-16	4.5 (0.17)	6.4 (0.20)	5.1 (0.18)	5.9 (0.20)	5.9 (0.19)	4.8 (0.23)	5.1 (0.32)	5.5 (0.13)
HPV-18	**3.9 (0.16)**	**6.2 (0.20)**	**4.7 (0.17)**	**5.4 (0.19)**	**5.6 (0.18)**	**4.6 (0.23)**	**4.0 (0.29)**	5.1 (0.13)
**Bacteria and Parasite**								
C.trachomatis	**47.6 (0.42)**	**51.0 (0.41)**	**48.6 (0.41)**	**50.1 (0.42)**	**55.1 (0.39)**	**44.0 (0.54)**	**39.0 (0.71)**	49.3 (0.29)
H.pylori	71.4 (0.38)	70.6 (0.37)	**63.5 (0.39)**	**78.8 (0.34)**	**70.9 (0.36)**	**71.7 (0.49)**	**70.1 (0.67)**	71.0 (0.27)
C.burnetii	11.0 (0.26)	11.0 (0.25)	10.4 (0.25)	11.7 (0.27)	11.4 (0.25)	10.3 (0.33)	11.1 (0.46)	11.0 (0.18)
T.gondii	22.6 (0.35)	22.0 (0.34)	**24.4 (0.35)**	**20.2 (0.33)**	**22.5 (0.33)**	**21.5 (0.45)**	**23.1 (0.61)**	22.3 (0.24)

Bold values denote statistical significance at the p<0.05 level.

BKV, BK polyomavirus; CMV, cytomegalovirus; EBV, Epstein-Barr virus; HBV, hepatitis B virus; HCV, hepatitis C virus;

HTLV, Human T-cell lymphotropic virus type 1; JCV, JC polyomavirus; MCV, Merkel cell polyomarvirus; VZV, varicella zoster virus.

eFigure 8. Seroprevalence (%) for each pathogen in each region, among incident cancer cases

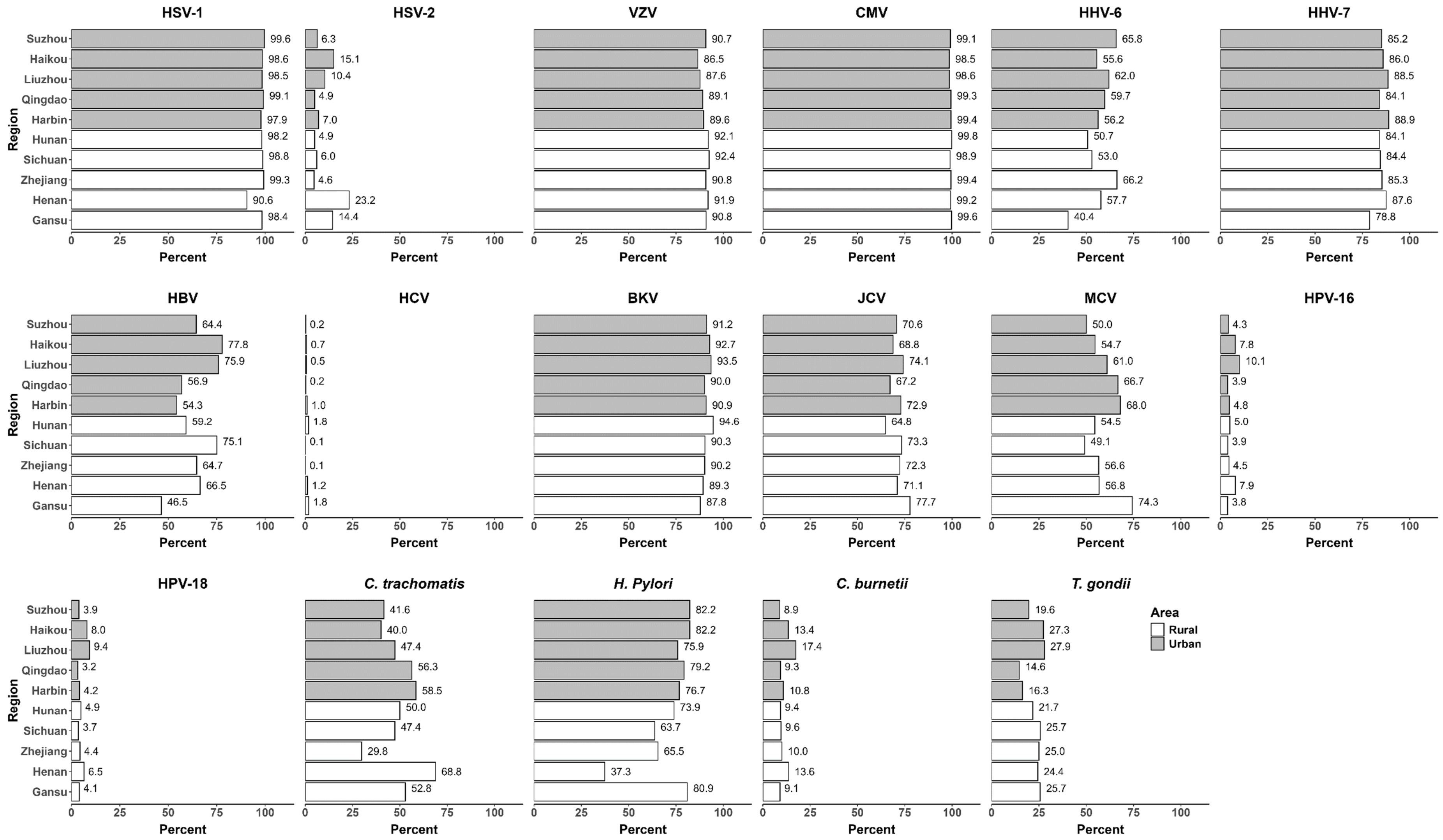

The grey bar represents urban participants and the white bar represents rural participants.

eFigure 9. Seroprevalence (%) of each pathogen by year of birth among incident cancer cases

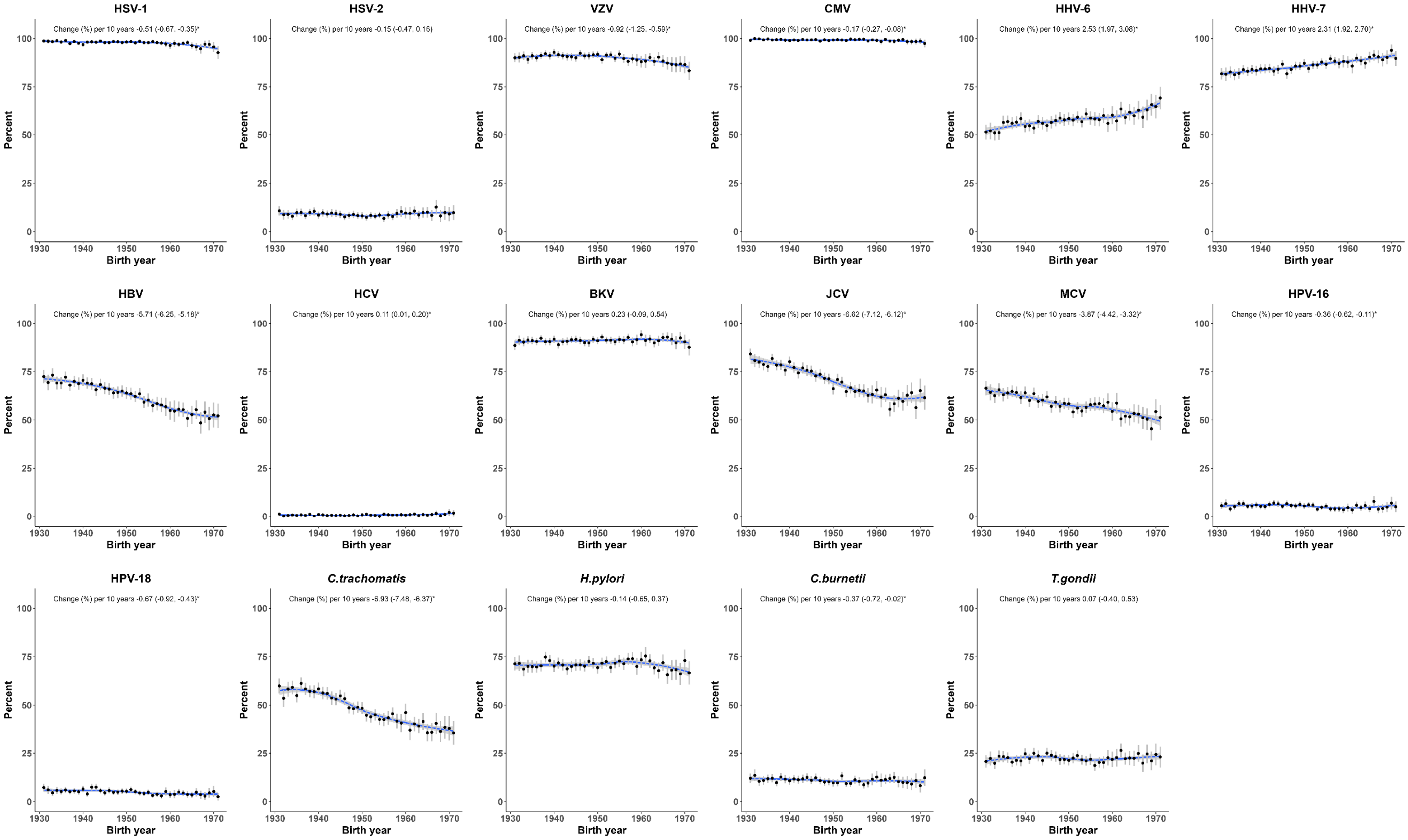

Dots and grey lines represent sero-prevalence estimates and 95% CI for every single birth year. The blue line represents estimates for these sero-prevalences derived from LOESS method, and the shaded areas show the 95% CI. Due to few participants that were born before 1932 or after 1971, those who were born between 1927 and 1931, or 1971 and 1976 were combined, separately.
LOESS, locally weighted scatterplot smoothing

eFigure 10. Prospective associations between the number of co-infected pathogens and risk of overall cancer incidence in Chinese adults

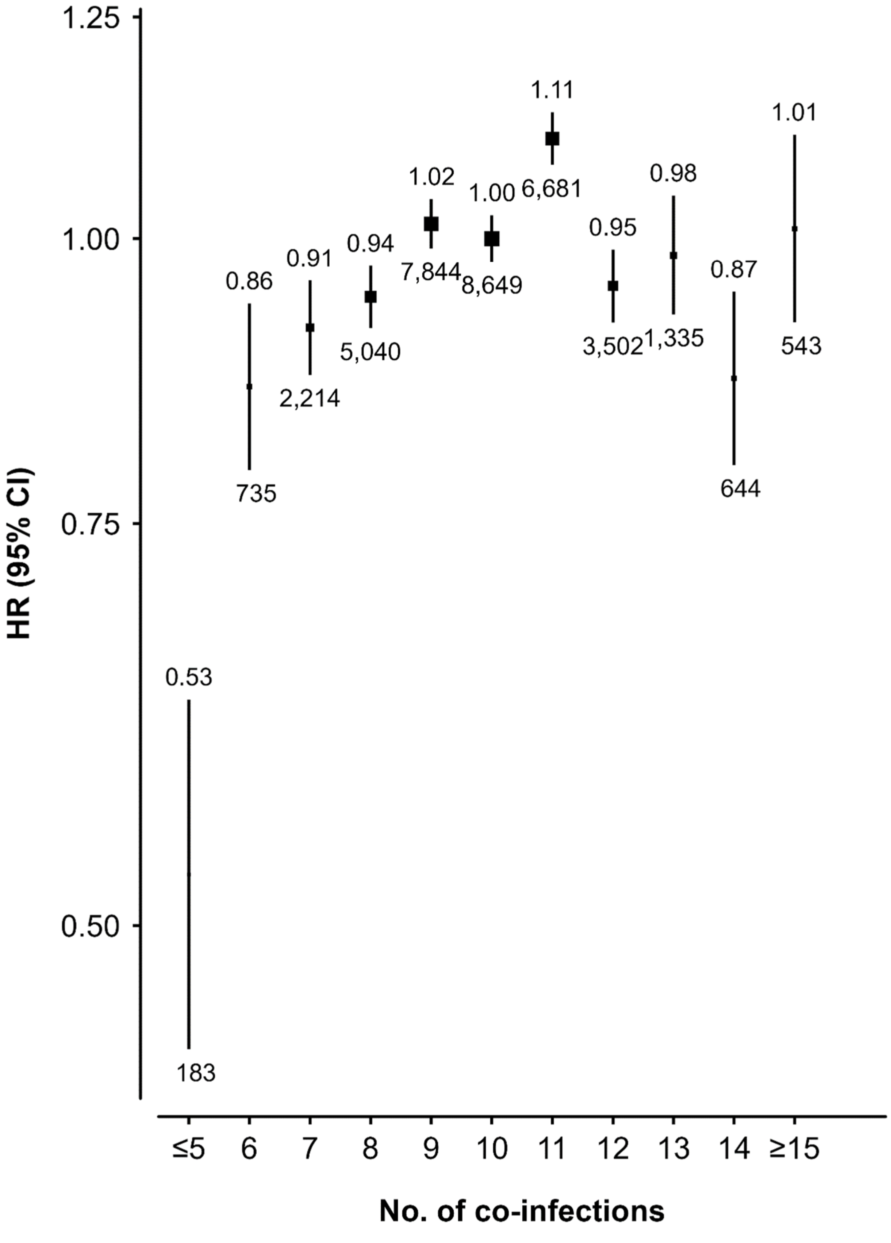

Adjusted HR for cumulative number of positive antigens were assessed using Cox proportional hazards models, using the Prentice pseudo-partial likelihood method. Models were adjusted for age, sex, region, education, smoking, alcohol, BMI and family history of cancer. The squares represent adjusted HRs with the area inversely proportional to the variance of the logHRs, and the vertical lines represent their corresponding 95% Cis.

eFigure 11. Adjusted HRs for cardia gastric cancer and non-cardia gastric cancer by *H. pylori*

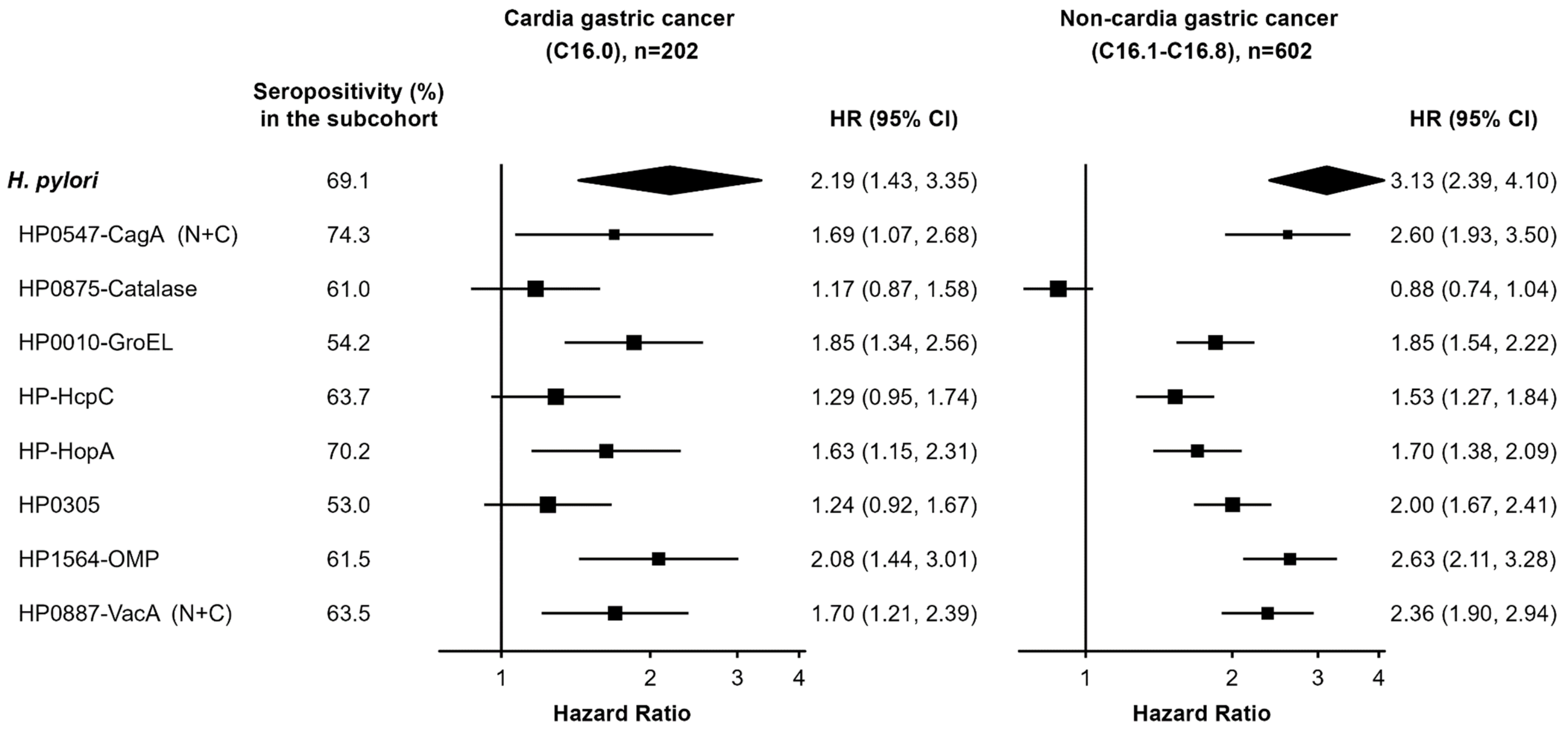

Cox proportional hazards models were fitted with the Prentice pseudo-partial likelihood method and the first two years' follow-up were excluded in the analyses. Models were adjusted for age, sex, study region, education, smoking, alcohol drinking, BMI and the family history of cancer, with time since recruitment as the underlying timescale. The diamonds represent adjusted HRs at pathogen level and the squares represent adjusted HRs at antigen level, both with the area inversely proportional to the variance of the logHRs, and the horizontal lines represent their corresponding 95% CIs.
